# Regulatory T cell frequencies and phenotypes following anti-viral vaccination

**DOI:** 10.1371/journal.pone.0179942

**Published:** 2017-06-28

**Authors:** A. Charlotte M. T. de Wolf, Susan van Aalst, Irene S. Ludwig, Caroline L. Bodinham, David J. Lewis, Ruurd van der Zee, Willem van Eden, Femke Broere

**Affiliations:** 1 Division of Immunology, Department of Infectious Diseases & Immunology, Utrecht University, Utrecht, The Netherlands; 2 Surrey Clinical Research Centre, University of Surrey, Guildford, United Kingdom; University of California Los Angeles, UNITED STATES

## Abstract

Regulatory T cells (Treg) function in the prevention of excessive inflammation and maintenance of immunological homeostasis. However, these cells may also interfere with resolution of infections or with immune reactions following vaccination. Effects of Treg on vaccine responses are nowadays investigated, but the impact of vaccination on Treg homeostasis is still largely unknown. This may be a relevant safety aspect, since loss of tolerance through reduced Treg may trigger autoimmunity.

In exploratory clinical trials, healthy adults were vaccinated with an influenza subunit vaccine plus or minus the adjuvant MF59^®^, an adjuvanted hepatitis B subunit vaccine or a live attenuated yellow fever vaccine. Frequencies and phenotypes of resting (rTreg) and activated (aTreg) subpopulations of circulating CD4^+^ Treg were determined and compared to placebo immunization.

Vaccination with influenza vaccines did not result in significant changes in Treg frequencies and phenotypes. Vaccination with the hepatitis B vaccine led to slightly increased frequencies of both rTreg and aTreg subpopulations and a decrease in expression of functionality marker CD39 on aTreg. The live attenuated vaccine resulted in a decrease in rTreg frequency, and an increase in expression of activation marker CD25 on both subpopulations, possibly indicating a conversion from resting to migratory aTreg due to vaccine virus replication.

To study the more local effects of vaccination on Treg in lymphoid organs, we immunized mice and analyzed the CD4^+^ Treg frequency and phenotype in draining lymph nodes and spleen. Vaccination resulted in a transient local decrease in Treg frequency in lymph nodes, followed by a systemic Treg increase in the spleen.

Taken together, we showed that vaccination with vaccines with an already established safe profile have only minimal impact on frequencies and characteristics of Treg over time. These findings may serve as a bench-mark of inter-individual variation of Treg frequencies and phenotypes following vaccination.

## Introduction

The immune system is trained to protect the host against a broad variety of pathogenic threats. When properly activated, a successful response to infection results in protection against the disease. However, to prevent excessive inflammation due to immune activation, the response needs to be tightly controlled to maintain and regain immunological homeostasis [1]. One of the mechanisms by which the body is equipped to do so, is via the negative control function of regulatory T cells (Treg) and improperly functioning Treg can result in severely uncontrolled immune reactions with autoimmunity and even death as result [2–4]. In addition to their role in prevention of autoimmunity, Treg were found to be imperative in the protection of the body from damage during (viral) infections. Several studies have shown that during infections the balance between functional Treg and effector T cells (Teff) is essential, as Treg depletion resulted in exacerbation of the disease, immune pathology and a delay in viral clearance [5–7]. On the other hand, Treg were also found to interfere in resolution of viral infections, e.g. in mouse models of hepatitis B virus (HBV) infection where their depletion led to more active, virus-specific T cells and improved viral clearance [8,9]. Thus, Treg regulation of immune responses can prevent autoimmunity and excessive tissue damage, but this process potentially allows persistent infections as well and therefore, the correct outcome of Treg dampening is vital [7,10].

Apart from infections, Treg may also interfere with immune reactions following vaccination. Dampening of the response to vaccines, which are usually mimics or derivatives of infectious agents, can potentially result in lower vaccine efficacy as was shown in anti-tumor vaccinations [11,12]. For this reason, the effect of Treg on vaccine responses is nowadays studied and several new strategies are explored to improve vaccine effectiveness by temporarily depleting or inhibiting Treg [13,14]. However, it has become more and more evident that the interaction between Treg and Teff during infection and vaccination is complex and pathogen-specific. Thus depletion of Treg might not be a straightforward solution to improve vaccine responses [10].

Not only knowledge on the effect of Treg on vaccine responses is essential, but also the other way around, namely the effect of vaccination on Treg requires attention. Because íf vaccination would significantly affect Treg frequency or function, this may result in uncontrolled (auto-)immune responses and thus be a relevant safety risk of vaccination. Therefore, we studied the effect of vaccinations on human CD4^+^ Treg responses, using a panel of four commonly used antiviral vaccines: a trivalent influenza vaccine (TIV) with and without the addition of adjuvant MF59^®^ (Fluad^®^ or Agrippal^®^), a HBV subunit vaccine (Engerix-B^®^) and a live attenuated yellow fever vaccine (Stamaril^®^). We analyzed peripheral blood mononuclear cells (PBMC) obtained from healthy volunteers that had been vaccinated in the context of broader study trials (BIOVACSAFE; [15]). At several days post vaccination (dpv), the frequency and phenotype of CD4^+^ Treg subpopulations in peripheral blood was examined by flow cytometry. For the analysis of local effects of the influenza and hepatitis B vaccines on Treg, we vaccinated mice and analyzed the CD4^+^ CD25^+^ Foxp3^+^ Treg frequency and phenotype in draining lymph nodes and spleen at several days post vaccination.

Here, we show that vaccination with commonly used, human anti-viral vaccines minimally impacts the frequency and characteristics of Treg over time in both men and mice, but that the effects are distinct for the different vaccines analyzed.

## Materials and methods

### Human inpatient study design

**Fig 1:**
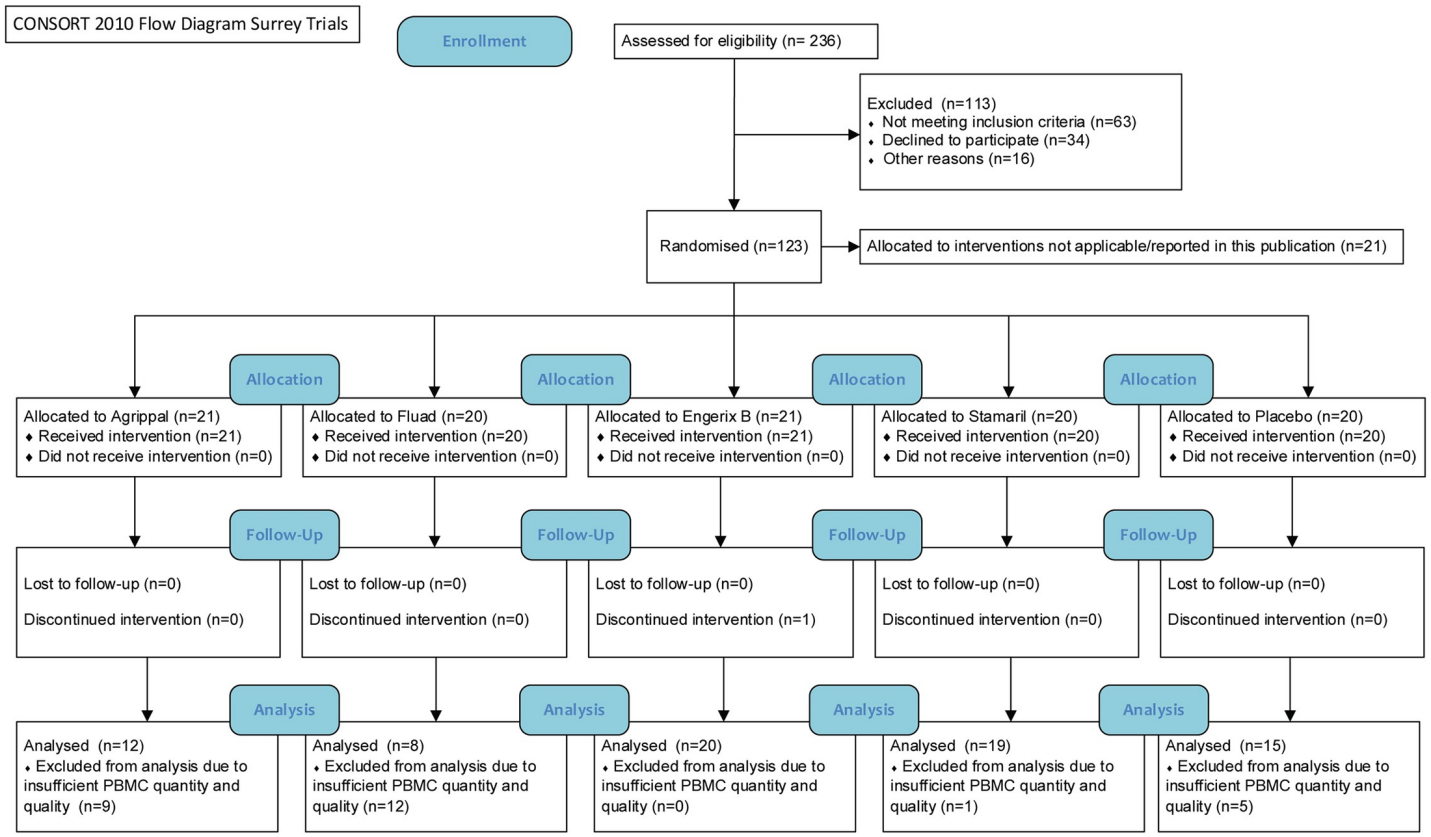
CONSORT flow diagram.

Three partial-blind (participant and laboratory), randomised, placebo-controlled exploratory studies (clinicaltrials.gov; NCT01765413, NCT01771354 and NCT01771367; protocols in S1–S3 Text and [15]) were conducted at the Surrey Clinical Research Centre, University of Surrey, Guildford, UK (as part of the BIOVACSAFE consortium-funded clinical study protocols) after approval of procedures by London-Surrey Borders Research Ethics Committee (REC References: 12LO/1871, 12/LO/1899, 13/LO/0044). The studies were undertaken in accordance with the Declaration of Helsinki and good clinical practices. Healthy participants (male and female) ranging from 18 to 45 years of age (characteristics in Table 1) were recruited into the studies after written informed consent. See CONSORT diagram and checklist (Fig 1 and S4 Text, respectively). Participants remained in the Centre from 1 day before vaccination until 5 days post vaccination for daily monitoring. After day 5, several outpatient follow-up visits took place.

**Table 1:** Summary of participant characteristics and pre-vaccination Treg values.

Parameter	Influenza vaccine	Hepatitis B vaccine	Yellow fever vaccine
***N* participants**			
Placebo	3	4	8
Vaccine	12 (Agrippal^®^)	20	19
Vaccine	8 (Fluad^®^)		
**Age** ^a^			
Placebo	33 ± 2.65	30 ± 3.63	29 ± 2.00
Vaccine	27 ± 2.06 (Agrippal^®^)	32 ± 1.47	30 ± 1.73
Vaccine	28 ± 2.05 (Fluad^®^)		
**Sex (M/F)** ^a^			
Placebo	1/2	2/2	5/3
Vaccine	3/9 (Agrippal^®^)	17/3	12/7
Vaccine	4/4 (Fluad^®^)		
**Baseline frequency (%)** ^a^^,^ ^b^			
CD4^+^CD45RA^+^FoxP3^+^ (rTreg)	1.02 ± 0.09	4.04 ± 0.41	3.01 ± 0.21
CD4^+^CD45RA^-^FoxP3^++^ (aTreg)	0.99 ± 0.10	2.32 ± 0.21	2.44 ± 0.18
**Baseline frequency (%) within rTreg** ^a^^,^ ^b^			
CD25	17.4 ± 1.1	13.5 ± 2.2	7.0 ± 0.9
CD39	23.0 ± 2.7	25.2 ± 2.0	16.1 ± 0.9
CD31	n.d.	85.8 ± 1.6	82.8 ± 1.3
HLA-DR	n.d.	52.5 ± 3.9	53.6 ± 1.5
CCR4	n.d.	51.8 ± 4.0	50.9 ± 1.9
CCR7	72.3 ± 3.5	63.5 ± 4.5	59.4 ± 2.1
**Baseline frequency (%) within aTreg** ^a^^,^ ^b^			
CD25	58.0 ± 1.5	49.9 ± 2.7	51.5 ± 1.7
CD39	67.1 ± 5.5	61.3 ± 5.6	66.3 ± 5.2
CD31	n.d.	17.8 ± 3.7	15.3 ± 1.4
HLA-DR	n.d.	47.5 ± 3.5	43.5 ± 2.2
CCR4	n.d.	76.7 ± 2.5	87.7 ± 1.1
CCR7	41.2 ± 2.5	23.3 ± 3.2	19.3 ± 1.6

^a^ mean ± SEM

^b^ for influenza and yellow fever vaccines baseline is day 0, for hepatitis B vaccine baseline is day 168

n.d. = not determined

#### Influenza

Participants, assumed to be previously primed with hemagglutinin via vaccination or natural infection, received either a single 0.5 ml dose of a non-adjuvanted TIV (Agrippal^®^, Novartis Vaccines), a MF59^®^-adjuvanted TIV (Fluad^®^, Novartis Vaccines) or placebo (saline). Both vaccines contained the recommended composition for the 2012/2013 northern hemisphere influenza season (as described in [16]). Vaccine or placebo was administered intramuscularly (i.m., *m*. *deltoideus*) and PBMC samples were collected just before vaccination (day 0) and at days 1, 3, 5, 14 and 21 after vaccination. PBMC were purified and cryopreserved until further processing.

#### Hepatitis B

Participants seronegative for anti-hepatitis B antibodies were vaccinated i.m. (*m*. *deltoideus*) with either 1 ml doses of an alum-adjuvanted hepatitis B vaccine (Engerix-B^®^ containing 20 μg hepatitis B surface antigen, GlaxoSmithKline) or placebo (saline). Vaccine or placebo doses were administered at days 0, 28 and 168 and PBMC samples were collected just before vaccination (day 168) and at day 1, 3 and 14 (days 169, 171 and 182) after dose 3. PBMC were purified and cryopreserved until further processing.

#### Yellow fever

Participants seronegative for anti-yellow fever antibodies were vaccinated subcutaneously (s.c., deltoid region) with a single 0.5 ml dose of a live attenuated yellow fever vaccine (Stamaril^®^ containing not less than 1000 IU, Sanofi Pasteur) or placebo (saline). Blood samples were collected just before vaccination (day 0) and at days 1, 5, 7 and 14 after vaccination. PBMC were purified and cryopreserved until further processing.

### Cell thawing

Vials containing frozen PBMC were quickly thawed in a 37°C water bath and cold RPMI medium (Gibco, Thermo Fisher Scientific, Landsmeer, The Netherlands) containing 5% NHS (Merck Millipore, Amsterdam, The Netherlands) was added dropwise to the cell suspension. The cells were washed twice with RPMI medium (5% NHS) and subsequently resuspended in PBS supplemented with 2% FBS (Lonza, Breda, The Netherlands) for flow cytometric staining.

### Animals

#### Ethical statement

All animal experiments were performed in strict accordance to the Dutch Animal Experimentation Act and EU directives 86/609/CEE and 2010/63/EU related to the protection of vertebrate animals used for experimental and other scientific purposes. The experimental protocols were approved by the Committee on Animal Experiments of the University of Utrecht (DEC2012.II.08.114) and performed in the Central Laboratory Animal Research Facility of the University of Utrecht, which has AAALAC (Association for Assessment and Accreditation of Laboratory Animal care) accreditation.

#### Mice

CB6F1 mice (female; 6–10 weeks) were obtained from Charles River Laboratories and were housed at the Central Laboratory Animal Research Facility of the University of Utrecht, The Netherlands. Mice were kept under standard conditions and received water and food *ad libitum*.

### Animal study design

Mice were randomly divided in control or treatment groups and at day 0, mice were injected i.m. (*m*. *quadriceps*) with 50 μl adjuvant/vaccine in PBS. Complete Freund’s Adjuvant (CFA, 0.5 mg/ml; Difco, New Jersey, USA) was prepared by mixing with equal volumes of PBS. Lipopolysaccharide (LPS; *E*. *coli* 0127:B8 L4516, Sigma-Aldrich, Zwijndrecht, The Netherlands) was injected as 0.5 mg/ml solution in 50 μl PBS. The influenza immunogens (TIV, a kind gift from Novartis Vaccines) consisted of 1.5 μg purified hemagglutinin and neuraminidase of each of the three influenza strains present in the Novartis Flu-vaccines as recommended for the 2012/2013 northern hemisphere influenza season (as described in [16]) in 50 μl PBS (TIV^−^) or in 25 μl PBS mixed with an equal volume of adjuvant MF59^®^ (Novartis Vaccines; TIV^+^). Hepatitis B immunization was performed with an injection of 50 μL of Engerix-B^®^ (20 μg/mL hepatitis B surface antigen, a kind gift from GlaxoSmithKline). The vaccine doses for mice equaled 1/10^th^ of a human vaccine dose. Mock-injected animals received 50 μl PBS.

All animals were monitored on a daily basis the first 2 days post immunization and from thereon weekly. No signs of pain or other distress were observed. At day 3, 7, 14 and 21 post vaccination animals were humanely euthanized using a carbon dioxide chamber and draining lymph nodes (dLN, *lnn*. *inguinales*) and spleen were removed and kept on ice in IMDM medium (Gibco, Thermo Fisher Scientific) supplemented with 2% FBS (Lonza) for further processing.

### Single cell suspensions

Single cell suspensions of draining lymph nodes and spleens were prepared using a 70 μm nylon mesh (BD Bioscience, New Jersey, USA). Erythrocytes were depleted from the splenic single cell suspension by incubation in ACK-lysis buffer (150 mM NH_4_Cl and 1 mM NaHCO_3_, pH 7.4) for 5 min at ice. Cells were resuspended in PBS supplemented with 2% FBS (Lonza) for flow cytometric staining.

### Flow cytometric analysis

For analysis of the human samples, all antibodies were purchased from Miltenyi Biotec (Leiden, The Netherlands), unless stated otherwise. Cells were surface-labeled with mouse-anti-human monoclonal antibodies CD4-APC (M-T466, IgG1), CD25-FITC (M-A251, IgG1, BD Biosciences), CD31-BV510 (WM59, IgG1, BD Biosciences), CD39-PE (MZ18-23C8, IgG1), CD45RA-PerCP (T6D11, IgG2b), HLA-DR-FITC (AC122, IgG2a), rat-anti-human monoclonal antibody CCR7-BV510 (3D12, IgG2a, BD Biosciences) and recombinant human monoclonal antibody CCR4-PE (REA279, IgG1). Intracellular Foxp3 staining (mouse-anti-human, 236A/E7, IgG1, eFluor450-labeled) was performed with a Foxp3 staining kit as instructed (eBioscience, Vienna, Austria).

Based on the surface expression of CD45RA and intranuclear expression of Foxp3, two phenotypically and functionally distinct Treg subsets within the CD4^+^ T cell population could be distinguished (as shown by [17]): resting/naïve regulatory T cells (rTreg) were defined as CD4^+^ CD45RA^+^ Foxp3^+^ and activated/effector regulatory T cells (aTreg) as CD4^+^ CD45RA^-^ Foxp3^++^. This gating strategy allowed us to exclude the activated non-suppressive effector T cells and analyze marker expression specifically within the Treg subsets. Natural Treg are defined as stable suppressive cells with a completely demethylated region in the FoxP3 gene. Within this population, resting/naïve Treg appear to be suppressive, but once activated can convert to activated/effector Treg with higher suppressive capacity [18]. The mean percentage of the respective human cell types and cell surface marker expression at day 0 is indicated in Table 1 and the used gating strategy is shown in S1 Fig.

For analysis of murine single cell suspensions, cells were stained with the monoclonal rat-anti-mouse antibodies CD4-V500 (RM4-5, IgG2a, BD Biosciences), CD25-PerCPCy5.5 (PC61.5, IgG1, eBioscience) and Armenian-hamster anti-mouse CD69-APC (H1.2F3, IgG, eBioscience). Intracellular Foxp3 staining (rat-anti-mouse, FJK-16s, IgG2a, eFluor450-labeled) was performed with a Foxp3 staining kit as instructed (eBioscience). Murine Treg were defined as CD4^+^ CD25^+^ Foxp3^+^ T cells.

Flow cytometry samples were measured on a Canto II (BD Biosciences) and analyzed using FlowJo software version 7.6.5 (Miltenyi Biotec).

### Statistical analysis

Per time point, a delta percentage of the Treg frequency was calculated and all data are represented as mean delta ± SEM. For the human data, the delta was determined as the percentage of Treg at a specific time point subtracted by the percentage at day 0 for the same donor (= % Treg day_x_—% Treg day_0_). In the case of the Engerix-B^®^ trial, day_0_ = day_168_, the moment just before the third immunisation. Differences between 2 groups were determined with an unpaired, two-tailed Mann-Whitney test. Differences between 3 groups were determined with the Kruskal-Wallis test.

For mice, the delta was determined as the percentage Treg at a specific time point subtracted by the mean percentage of the mock-injected animals at that time point (= % Treg treatment day_x_—average % Treg PBS day_x_). Differences between 2 groups were determined with an unpaired two-tailed student’s t-test with Welch’s correction. Differences between 3 or more groups were determined with a one-way ANOVA (two-tailed) with Dunnett’s multiple comparison test.

Statistical analysis was performed using Prism 6 (GraphPad software). P < 0.05 was considered significant.

## Results

### Treg responses following vaccination in humans

To test whether vaccination leads to changes in the circulating Treg population, healthy individuals were i.m. or s.c. injected with a non-adjuvanted TIV (Agrippal^®^), a MF59^®^-adjuvanted TIV (Fluad^®^), an alum-adjuvanted hepatitis B vaccine (Engerix-B^®^), a live attenuated yellow fever vaccine (Stamaril^®^) or placebo. Treg frequency and characteristics were monitored right before and at different time points after vaccination.

#### Influenza

**Fig 2:**
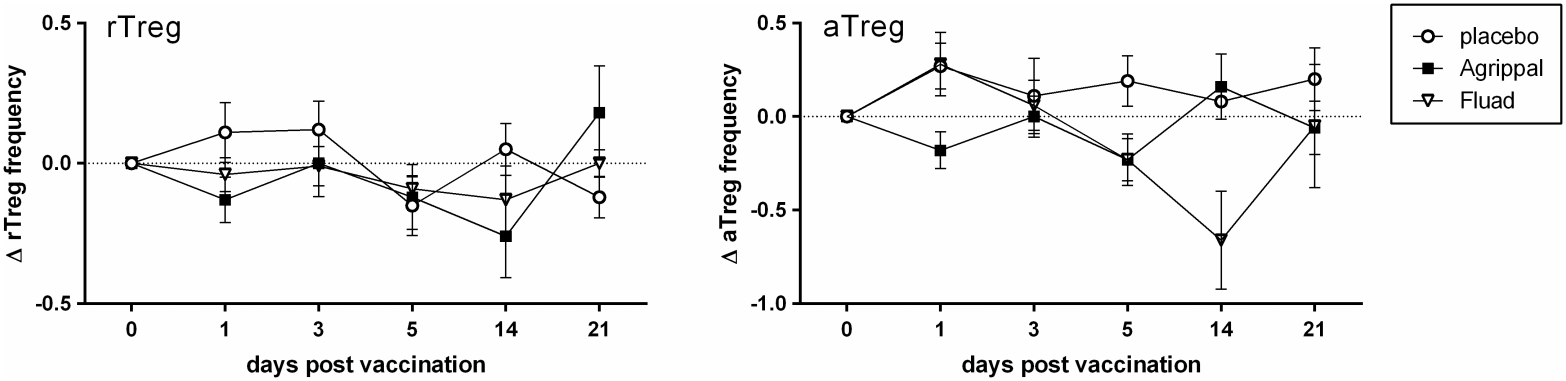
Effect of vaccination of humans with subunit influenza vaccines on Treg frequency. At day 0, healthy adults were i.m. vaccinated with a non-adjuvanted (Agrippal^®^) or MF59^®^-adjuvanted (Fluad^®^) trivalent influenza subunit vaccine or injected with a placebo. At day 1, 3, 5, 14 and 21 post vaccination changes in Treg frequency were determined. The delta Treg percentage per time point was determined per donor (= % Treg day_x_—% Treg day_0_). Mean (± SEM) delta percentage of the frequency of rTreg (left) and aTreg (right) in the blood at the different time points after vaccination with Agrippal^®^ (n = 12), Fluad^®^ (n = 8, day 14 n = 2) or placebo (n = 6). Means were statistically compared with a Kruskal-Wallis test.

After vaccination with Agrippal^®^ and Fluad^®^, we observed a small, but non-significant decrease in rTreg frequency shortly after vaccination (day 1) and also two weeks later (day 14) compared with placebo (Fig 2). During the first 5 dpv with Agrippal^®^, the aTreg frequency was slightly lower compared to placebo injection and equal to pre-vaccination values again around 14 dpv. Fluad^®^ vaccination led to a decrease in aTreg frequencies at 5 and 14 dpv, though this did not reach statistical significance (Fig 2).

To further dissect the characteristics within the Treg subpopulations, we analyzed the expression of several markers related to Treg functionality and migration (Table 1). After vaccination with Agrippal^®^ and Fluad^®^, CD25 expression was lower (trend) on both rTreg (Figure A in S2 Fig) and aTreg (Figure B in S2 Fig) during the first 5 dpv compared to placebo injection. This was in contrast with the expression of CD39 and CCR7 on the rTreg subset, which was increased during this period (Figure A in S2 Fig). Compared with the placebo-injected group, vaccination with the influenza vaccines did not result in remarkable changes in the percentage of CD39^+^ or CCR7^+^ aTreg (Figure B in S2 Fig).

Taken together, vaccination with a non-adjuvanted or MF59^®^-adjuvanted subunit influenza vaccine only led to minor changes in rTreg and aTreg frequencies and characteristics, most of these shortly after vaccination.

#### Hepatitis B

As different vaccination strategies may result in different responses in the Treg population, we tested another frequently used subunit vaccine, the hepatitis B vaccine Engerix-B^®^, for its effect on Treg. This immunization requires a three-dose schedule to induce a protective antibody titer. Since antibody responses became only significantly measurable after the third dose, we decided to focus on changes in Treg frequency after this dose (day 168).

**Fig 3:**
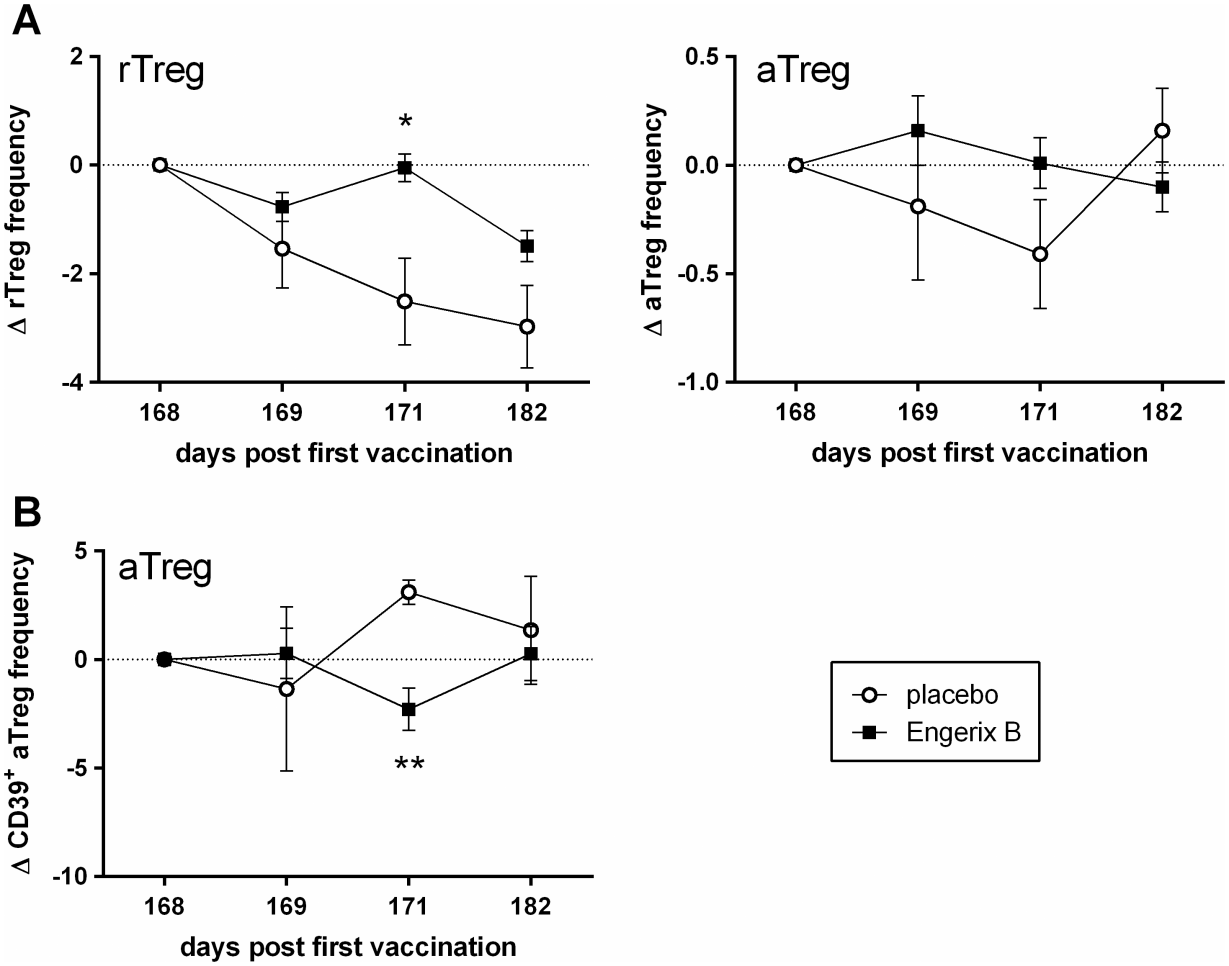
Effect of vaccination of humans with a subunit hepatitis B vaccine on Treg frequency and characteristics. At day 0, day 28 and day 168, healthy adults were i.m. vaccinated with Engerix-B^®^ or injected with a placebo. At day 1, 3, and 14 after the third immunization dose (day 169, 171 and 182) changes in Treg frequency and phenotype were determined. The delta Treg percentage per time point was determined per donor (= % Treg day_x_—% Treg day_168_). **(A)** Mean (± SEM) delta percentage of the frequency of rTreg (left) and aTreg (right) in the blood at the different time points after the third dose of Engerix-B^®^ (n = 20) or placebo (n = 4). **(B)** Mean (± SEM) delta percentage of CD39 expression on aTreg after vaccination. Means were statistically compared with an unpaired, two-tailed Mann-Whitney test (* p < 0.05, ** p < 0.01, relative to placebo at the same time point).

Vaccination with Engerix-B^®^ led to an initial decrease in rTreg frequency, followed by a significant increase 3 dpv (day 171) compared with control participants. Hereafter, the frequency decreased again but it remained higher after Engerix-B^®^ vaccination than placebo injection (Fig 3A). In contrast to rTreg, the frequency of the aTreg population increased the first day after Engerix-B^®^ vaccination but was not statistically different from placebo (Fig 3A). In general, in the placebo group we observed a decrease in Treg frequency (both rTreg and aTreg).

Effect of Engerix-B^®^ vaccination on Treg phenotype was most obvious in the aTreg population. In this population, expression of CD25 was first increased compared to placebo (day 169; trend), but then decreased again (day 171; Figure B in S3 Fig). Furthermore, CD31 appeared to be upregulated after vaccination (Figure B in S3 Fig), whereas CD39 (Fig 3B; significant) and HLA-DR (Figure B in S3 Fig; trend) both decreased during the first days after vaccination.

With respect to the rTreg population, no significant differences in surface marker expression between vaccinated and placebo-injected participants were observed (Figure A in S3 Fig). Nevertheless, an increase in CCR7 expression on rTreg was observed on the first day after vaccination compared to placebo which was followed by a decreased CCR7 expression (Figure A in S3 Fig; trend). A similar, though less pronounced effect of Engerix-B^®^ was observed for CCR7 expression on aTreg (Figure B in S3 Fig).

Overall, hepatitis B immunization with a three-dose schedule of Engerix-B^®^ resulted in significant increases in rTreg frequency as well as in significant changes in aTreg CD39 expression.

#### Yellow fever

Since live vaccines mimic a natural infection more closely than subunit vaccines, they may have a more pronounced effect on Treg. We therefore determined the frequency and surface marker expression on resting and activated Treg in yellow fever-naïve individuals before and in the first two weeks after vaccination with a live attenuated vaccine (Stamaril^®^).

**Fig 4:**
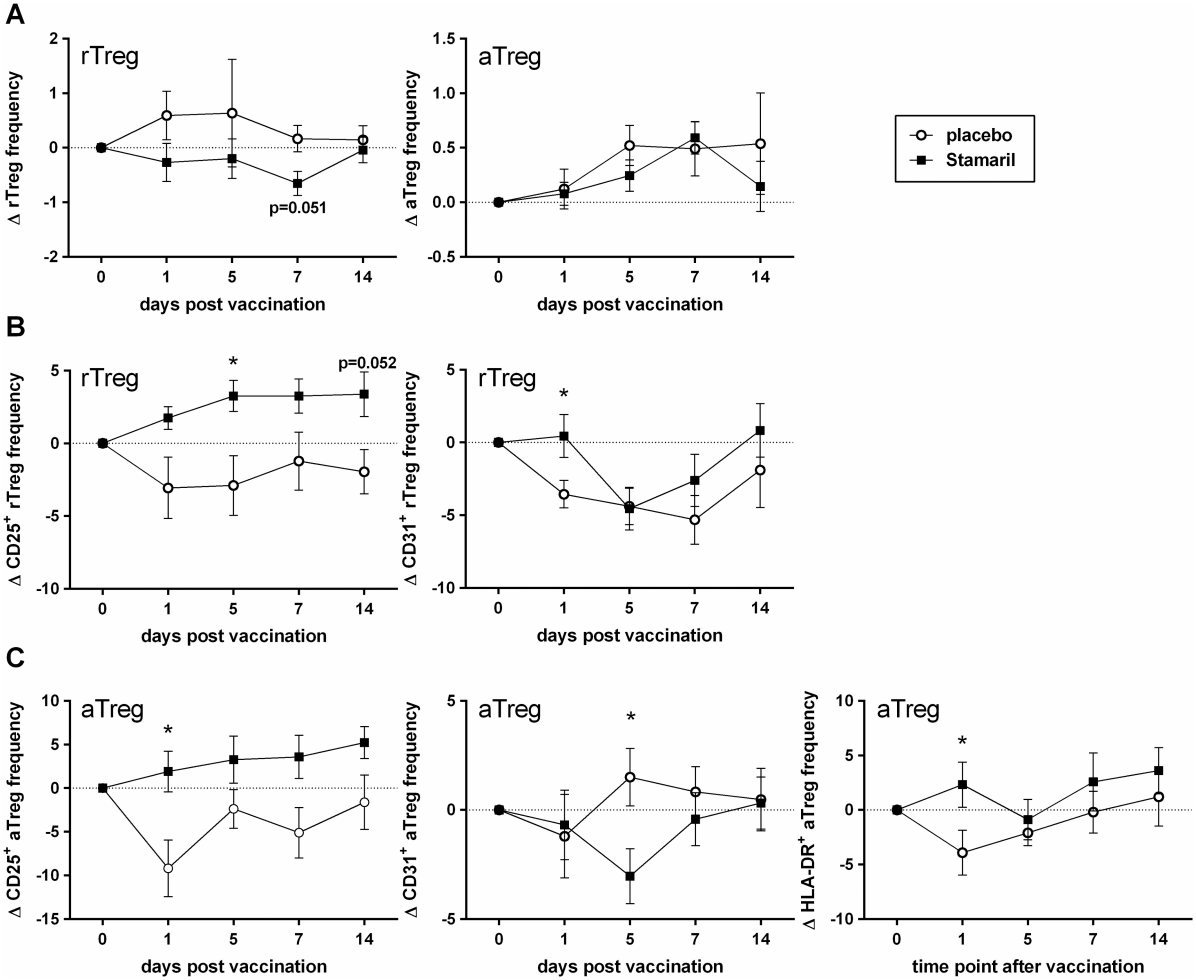
Effect of vaccination of humans with a live attenuated yellow fever vaccine on Treg frequency and characteristics. At day 0, healthy adults were s.c. vaccinated with Stamaril^®^ or injected with a placebo. At day 1, 5, 7 and 14 post vaccination changes in Treg frequency and phenotype were determined. The delta Treg percentage per time point was determined per donor (= % Treg day_x_—% Treg day_0_). **(A)** Mean (± SEM) delta percentage of the frequency of rTreg (left) and aTreg (right) in the blood at the different time points after vaccination with Stamaril^®^ (n = 19) or placebo (n = 8). **(B)** Mean (± SEM) delta percentage of CD25 and CD31 expression on rTreg after vaccination. **(C)** Mean (± SEM) delta percentage of CD25, CD31 and HLA-DR expression on aTreg after vaccination. Means were statistically compared with an unpaired, two-tailed Mann-Whitney test (* p < 0.05 relative to placebo at the same time point).

Compared to placebo injection, yellow fever vaccination led to a decrease in rTreg frequencies, which returned to the pre-vaccination values 14 dpv (Fig 4A). The aTreg showed an opposite trend, with an increase until 7 dpv and a return to the pre-vaccination values around 14 dpv (Fig 4A). The vaccine-induced changes in Treg frequency coincided with a significant increase in the expression of CD25 on rTreg as well as on aTreg (Fig 4B and 4C). Furthermore, HLA-DR expression on aTreg and CD31 expression on rTreg was significantly higher after Stamaril^®^ vaccination compared to placebo injection (Fig 4B and 4C), but CD31 expression on aTreg significantly dipped at 5 dpv (Fig 4C). A (small) dip in frequency at day 5 was also observed for expression of HLA-DR and CD31 on rTreg and aTreg and for CCR4 and CCR7 on rTreg (Fig 4B and 4C and Figure A and B in S4 Fig).

At 14 dpv, when the frequency of rTreg in the peripheral blood had returned to normal, expression of CD39, CCR4 and CCR7 on the rTreg was increased in the vaccinated group compared to the placebo group (Figure A in S4 Fig). Vaccination with Stamaril^®^ did not affect CCR4 or CCR7 expression on aTreg (Figure B in S4 Fig).

Taken together, our results revealed that administration of a live attenuated yellow fever vaccine induced changes in Treg frequencies and activation over time.

### Treg responses following vaccination in mice

Since we could not determine the local effects of vaccination in humans, we analyzed the effects of anti-viral vaccination on CD4^+^ CD25^+^ Foxp3^+^ Treg in mice. For that purpose, mice were injected (i.m.) with the subunit vaccines TIV^−^, TIV^+^ and Engerix-B^®^. LPS, known to affect Treg, was included as positive control, as was the strong immunogenic adjuvant CFA [19,20].

**Fig 5:**
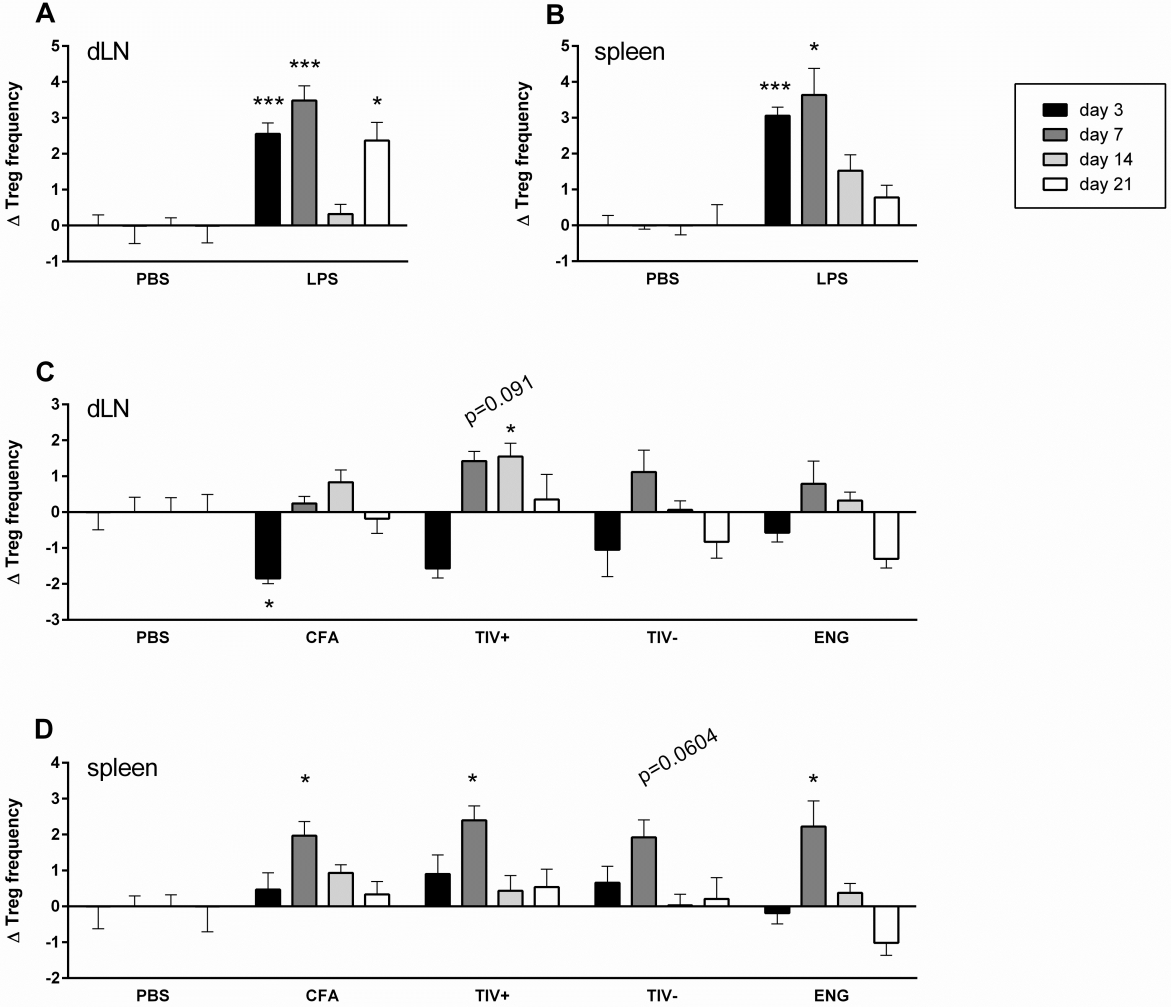
Effect of vaccination on local and systemic Treg frequency in mice. At day 0, mice were i.m. injected with LPS, CFA or with one of the vaccines (TIV^+^, TIV^-^ and Engerix-B^®^). Mock-injected animals received PBS. At day 3, 7, 14 and 21 post vaccination changes in Treg frequency were determined. The delta Treg percentage per time point was determined by comparing the treatment with the mean of PBS (= % Treg treatment day_x_—average % Treg PBS day_x_). The mean percentage Treg in PBS-injected mice was 10.9 ± 1.4% (dLN) and 12.0 ± 1.5% (spleen). N = 3–7 mice per group. **(A-D)** Mean (± SEM) delta percentage of Treg (CD4^+^ CD25^+^ Foxp3^+^) frequency at the different time points after vaccination were analyzed in dLN (A and C) and spleen (B and D) after LPS injection (A and B) and CFA or vaccine injection (C and D). Differences between PBS and LPS (A and B) were determined with an unpaired two-tailed student’s t-test with Welch’s correction. Differences between PBS and vaccines (C and D) were determined with a one-way ANOVA followed by Dunnett’s multiple comparisons test (* p < 0.05, ** p < 0.01, *** p < 0.001 relative to PBS at the same time point). TIV^+^: TIV supplemented with MF59^®^ adjuvant; TIV^-^: TIV only; ENG: Engerix-B^®^

Both in the dLN and in the spleen, LPS injection induced an increase in Treg frequency that was most evident at 3 and 7 dpv (Fig 5A and 5B). Injection of CFA, and, to a lesser extent, TIV^+^, TIV^−^and Engerix-B^®^ resulted in a decrease in Treg frequency in the dLN at 3 dpv (Fig 5C), which was accompanied by a decrease in the expression of activation marker CD69 on Treg (S5 Fig). However, subsequently, from 7 to 14 dpv the Treg frequency in dLN after vaccination returned to levels similar or even higher than after mock injection. A systemic effect of immunization could be observed at 7 dpv, when the Treg frequency in the spleen was (significantly) increased for CFA and the three vaccines compared to mock-injected animals (Fig 5D). This was followed by the re-establishment of original Treg frequencies.

Thus, vaccination of mice resulted locally in an early, transient decrease in Treg frequency and activation, subsequently followed by a systemic Treg increase.

## Discussion

In the current study, we have determined the effect of different anti-viral vaccinations on Treg responses. To the best of our knowledge, the present placebo-controlled study is the first in reporting this effect on the kinetics of frequency and characteristics of Treg subpopulations in healthy individuals.

We showed that vaccination of healthy individuals with a non-adjuvanted (Agrippal^®^) or MF59^®^-adjuvanted (Fluad^®^) influenza vaccine resulted only in minor changes in rTreg and aTreg frequencies, not significantly different from each other nor from placebo (Fig 2). Others have reported that vaccination with a different non-adjuvanted influenza vaccine also did not result in significant Treg frequency changes in healthy individuals, although significant increases were found in vaccinated rheumatoid arthritis patients in the first month following vaccination [21]. In contrast to vaccination, infection with an influenza virus was shown to increase Treg frequencies or absolute numbers in human peripheral blood and influenza A virus infection of mice induced a viral-specific Treg response locally as well as systemically that preceded the Teff response [22–25]. This shows that the influenza vaccines used in the current study have a much smaller effect on Treg than natural infections have.

Vaccination with another anti-viral subunit vaccine, Engerix-B^®^, did result in significant changes in Treg frequency. Compared to placebo, we found a significant increase in rTreg frequency at 3 dpv (day 171) after an initial decrease at 1 dpv (day 169) (Fig 3A). Other studies reported that in most cases hepatitis B vaccination or chronic HBV infection increased Treg frequencies in peripheral blood, potentially impairing vaccine effectiveness or viral clearance and reducing the response to treatment [8,26–31]. These studies, however, did not analyze frequency and marker expression within the different Treg subpopulations, except for the study from Mathew and colleagues [26]. This study found a negative correlation between the rTreg population and HBV-specific titres, although this was only found in hemodialysis patients and not in the healthy cohort. Nevertheless, this indicates that changes in Treg subpopulations are (at least in part) responsible for insufficiently protective immune responses following HBV vaccination, mainly in individuals with immune-related disorders. Of possible interest in this respect, studies have hypothesized relationships between HBV vaccination and autoimmunity (reviewed by Perricone and colleagues [32]).

The most obvious change in the aTreg population after Engerix-B^®^ vaccination was a dip in CD39 expression at 3 dpv (day 171; Fig 3B). This finding may indicate a difference in Treg functionality or an efflux of CD39^+^ effector/memory-like Treg, as CD39 expression positively correlates with Foxp3 levels and is involved in the immune suppression during HBV infection [33–36]. Loss of activated cells could also explain the decrease in CD39^+^ Treg, as these effector cells are prone to apoptosis [18]. This, however, will need further investigation.

We also determined the effect of a live attenuated yellow fever vaccine on rTreg and aTreg. Our results show an activation of rTreg with potential conversion to aTreg. This is demonstrated by a significant increase of CD25 expression on rTreg (Fig 4B) and a decrease in frequency of this subpopulation (Fig 4A). Blom and colleagues [37] also found an increase in Treg activation after yellow fever vaccination and another study from Martins and colleagues [38] observed a decrease in Treg frequency, though both studies looked in a differently defined Treg population. Possibly, replication of the vaccine virus activated the rTreg that subsequently converted to aTreg with a potentially increased functionality (based on a trend towards higher CD39 expression, Figure B in S4 Fig) [18]. This indicates that the effects on Treg after vaccination with a live attenuated virus resemble more closely a natural infection than do the subunit vaccines.

Remarkably, all vaccines showed a trend of increased CCR7 expression on rTreg (S2, S3 and S4 Figs). This increase might be a common feature of vaccinations indicative of an influx of naïve (CD45RA^+^ CCR7^+^) rTreg that migrate between secondary lymphoid organs and peripheral blood [39,40]. Together with an increased expression of CCR4, especially after yellow fever vaccination (Figure A in S4 Fig), this would indicate that naïve rTreg that entered the peripheral blood were preparing for migration towards and interaction with activated antigen-presenting cells [38,41].

Phenotypic characterization of Treg after vaccination is a first step to analyze the effect of vaccination on Treg and functional assays would be of value in this work. However, we had to limit our analysis to phenotypic characterization for several reasons. In general, the % Treg in blood is low and *ex vivo* expansion of Treg to enhance the yield of Treg per donor was no option since Treg can change both phenotypically and functionally by culturing (personal experience and [42]). Furthermore, because of the set-up of this study, participants gave blood with a high frequency and thus were the drawn blood samples too small to yield sufficient Treg for such assays.

Local effects of vaccination are difficult to determine in humans and we therefore assessed this in mice. When i.m. injecting LPS, we observed transient local and systemic increases in Treg frequency (Fig 5A and 5B). This corresponds with the tuned suppression model: upon pathogenic encounter (and thus presence of TLR ligands), Teff expand and produce interleukin-2, which in turn induces Treg expansion [19]. Pathogenic clearance allows for suppression of Teff by the expanded Treg and while interleukin-2 levels go down, so do the (relative) Treg numbers [1,19].

In contrast to LPS injection, immunization with CFA, and to a lesser extent vaccination with the three subunit vaccines, led to a local dip in Treg frequencies in the dLN at 3 dpv, which was followed by a systemic increase in the spleen at 7 dpv (Fig 5C and 5D). This early local decrease in Treg frequency probably does not represent an actual decrease in Treg number, but rather is the result of a vaccination-induced influx of other cells (e.g. Teff, or neutrophils/monocytes) in the dLN [43,44]. Indeed, when we considered actual cell numbers in the dLN, we found an increase in total numbers of cells in the dLN at 3 and 7 dpv (Figure A in S6 Fig), corresponding to (small) increases in numbers of CD4^+^ T cells and also CD4^+^ CD25^+^ Treg (Figure B and C in S6 Fig). These local (and systemic) increases in Treg numbers roughly correspond with Treg responses after infection, e.g. sub-lethal influenza virus infection induces increases in Treg numbers in the dLN and spleen 5 days post infection [25].

Noteworthy, the systemic changes in Treg frequency found in mice were not identical to the human data. This may be caused by the fact that the human systemic Treg frequency was determined in blood and that of mice in the spleen or caused by interspecies differences in Treg definitions.

## Concluding remarks

Although vaccines are usually regarded as mimics of infection, the present study has shown that inactivated (subunit) vaccines have only a minor effect on Treg frequencies, even in the presence of an adjuvant. The safe and widely used live attenuated vaccine closer resembled an infection with a more similar effect on frequency, activation and migration of the Treg. It therefore appears that Treg frequencies and phenotypes can fluctuate to a certain extent without causing a safety concern. However, to what extent vaccines also change Treg suppressive capacity and their characteristics will require further investigation, preferably in humans.

## Supporting information

S1 Fig Gating strategy of resting and activated Treg and expression of phenotypic markers. Representative flow cytometry plots of a placebo-injected individual to demonstrate gating strategy. This example belongs to a participant from the placebo group of the Stamaril trial (day_0_) and is representative for the overall gating. **(A)** In the fixed lymphocyte cell population, CD4^+^ T cells are selected and within this population rTreg (CD45RA^+^Foxp3^+^) and aTreg (CD45RA^-^Foxp3^++^) are defined. **(B)** Gating strategy of phenotypic marker expression on rTreg and aTreg for the markers CD25, CD39, CD31 (panel 1) and HLA-DR, CCR4 and CCR7 (panel 2). Gates were set on the CD45RA^+^Foxp3^-^ non-Treg population and were subsequently placed on the rTreg and aTreg cell populations to determine the expression of these markers.
(TIF)

S2 Fig Effect of vaccination of humans with subunit influenza vaccines on Treg characteristics. At day 0, healthy adults were i.m. vaccinated with a non-adjuvanted (Agrippal^®^) or MF59^®^-adjuvanted (Fluad^®^) trivalent influenza subunit vaccine or injected with a placebo. At day 1, 3, 5, 14 and 21 post vaccination changes in Treg frequency and phenotype were determined. The delta Treg percentage per time point was determined per donor (= % Treg day_x_—% Treg day_0_). **(A)** Mean (± SEM) delta percentage of CD25, CD39 and CCR7 expression on rTreg after vaccination (top to bottom). **(B)** Mean (± SEM) delta percentage of CD25, CD39 and CCR7 expression on aTreg after vaccination (top to bottom). Means were statistically compared with a Kruskal-Wallis test.
(TIF)

S3 Fig Effect of vaccination of humans with a subunit hepatitis B vaccine on Treg characteristics. At day 0, day 28 and day 168, healthy adults were i.m. vaccinated with Engerix-B^®^ or injected with a placebo. At day 1, 3, and 14 after the third immunization dose (day 169, 171 and 182) changes in Treg frequency and phenotype were determined. The delta Treg percentage per time point was determined per donor (= % Treg day_x_—% Treg day_168_). **(A)** Mean (± SEM) delta percentage of CD25, CD39, CD31, HLA-DR, CCR4 and CCR7 expression on rTreg after vaccination. **(B)** Mean (± SEM) delta percentage of CD25, CD31, HLA-DR, CCR4 and CCR7 expression on aTreg after vaccination. Means were statistically compared with an upaired, two-tailed Mann-Whitney test.
(TIF)

S4 Fig Effect of vaccination of humans with a live attenuated yellow fever vaccine on Treg characteristics. At day 0, healthy adults were s.c. vaccinated with Stamaril^®^ or injected with a placebo. At day 1, 5, 7 and 14 post vaccination changes in Treg frequency and phenotype were determined. The delta Treg percentage per time point was determined per donor (= % Treg day_x_—% Treg day_0_). **(A)** Mean (± SEM) delta percentage of CD39, HLA-DR, CCR4 and CCR7 expression on rTreg after vaccination. **(B)** Mean (± SEM) delta percentage of CD39, CCR4 and CCR7 expression on aTreg after vaccination. Means were statistically compared with an unpaired, two-tailed Mann-Whitney test.
(TIF)

S5 Fig Effect of vaccination in dLN on Treg CD69 expression in mice. At day 0, mice were i.m. injected with CFA or with one of the vaccines (TIV^+^, TIV^-^ and Engerix-B^®^). Mock-injected animals received PBS. At day 3, 7, 14 and 21 post vaccination changes in CD69 expression on Treg were determined in the dLN. The delta CD69 percentage per time point was determined by comparing the treatment with the mean of PBS (= % CD69 treatment day_x_—average %CD69 PBS day_x_). The mean percentage CD69 in PBS-injected mice was 27.6 ± 4.5%. Mean (± SEM) delta percentage are indicated. N = 3–7 mice per group. Differences were determined with a one-way ANOVA followed by Dunnett’s multiple comparisons test (* p < 0.05, ** p < 0.01 relative to placebo at the same time point). TIV^+^: TIV supplemented with MF59^®^ adjuvant; TIV^-^: TIV only; ENG: Engerix-B^®^.
(TIF)

S6 Fig Effect of vaccination in dLN on total cell numbers, CD4^+^ T cell and Treg numbers in mice. At day 0, mice were i.m. injected with LPS, CFA or with one of the vaccines (TIV^+^, TIV^-^ and Engerix-B^®^). Mock-injected animals received PBS. At day 3, 7, 14 and 21 post vaccination changes in numbers of **(A)** total cells, **(B)** CD4^+^ T cells and **(C)** Treg in the dLN were determined. The delta value per time point was determined by comparing the treatment with the mean of PBS (= number of cells (treatment) day_x_—average number of cells (PBS) day_x_). The mean number of cells in PBS-injected mice was 7.3x10^5^ ± 3.7x10^5^ (total cell numbers), 3.0x10^5^ ± 1.6x10^5^ (CD4^+^ T cells) and 3.3x10^5^ ± 1.9x10^5^ (Treg). Mean (± SEM) delta cell numbers are indicated. N = 3–7 mice per group. TIV^+^: TIV supplemented with MF59^®^ adjuvant; TIV^-^: TIV only; ENG: Engerix-B^®^.
(TIF)

S1 Text Clinical trial protocol influenza. (PDF)

S2 Text Clinical trial protocol hepatitis B. (PDF)

S3 Text Clinical trial protocol yellow fever. (PDF)

S4 Text CONSORT checklist (for three clinical trials). (DOC)

## Abbreviations

aTreg: activated regulatory T cell(s)
CFA: Complete Freund’s Adjuvant
dLN: draining lymph node(s)
dpv: days post vaccination
FBS: fetal bovine serum
HBV: hepatitis B virus
i.m.: intramuscular(ly)
LPS: lipopolysaccharide
NHS: normal human serum
PBMC: peripheral blood mononuclear cell(s)
rTreg: resting regulatory T cell(s)
s.c.: subcutaneous(ly)
Teff: effector T cell(s)
TIV: trivalent influenza vaccine
Treg: regulatory T cell(s)
