# Supplementary figures and images for: Regulatory T cell frequencies and phenotypes following anti-viral vaccination
Source: PLoS One. 2017 Jun 28;12(6):e0179942. doi: 10.1371/journal.pone.0179942 (PMC5489208; doi:10.1371/journal.pone.0179942)

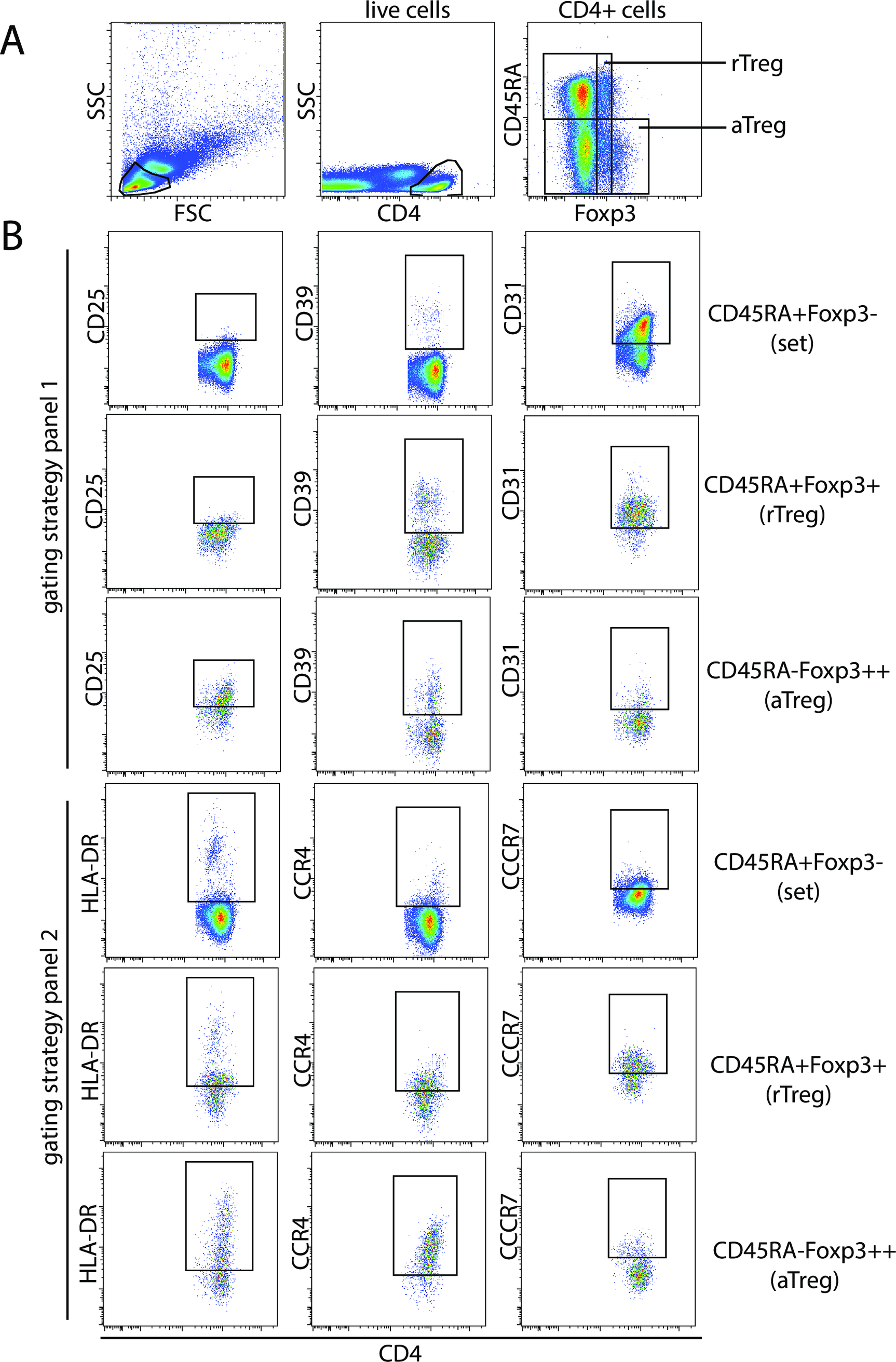

Supplement: S1 Fig — Representative flow cytometry plots of a placebo-injected individual to demonstrate gating strategy. This example belongs to a participant from the placebo group of the Stamaril trial (day0) and is representative for the overall gating. (A) In the fixed lymphocyte cell population, CD4+ T cells are selected and within this population rTreg (CD45RA+Foxp3+) and aTreg (CD45RA-Foxp3++) are defined. (B) Gating strategy of phenotypic marker expression on rTreg and aTreg for the markers CD25, CD39, CD31 (panel 1) and HLA-DR, CCR4 and CCR7 (panel 2). Gates were set on the CD45RA+Foxp3- non-Treg population and were subsequently placed on the rTreg and aTreg cell populations to determine the expression of these markers. (TIF) [file pone.0179942.s001.tif]

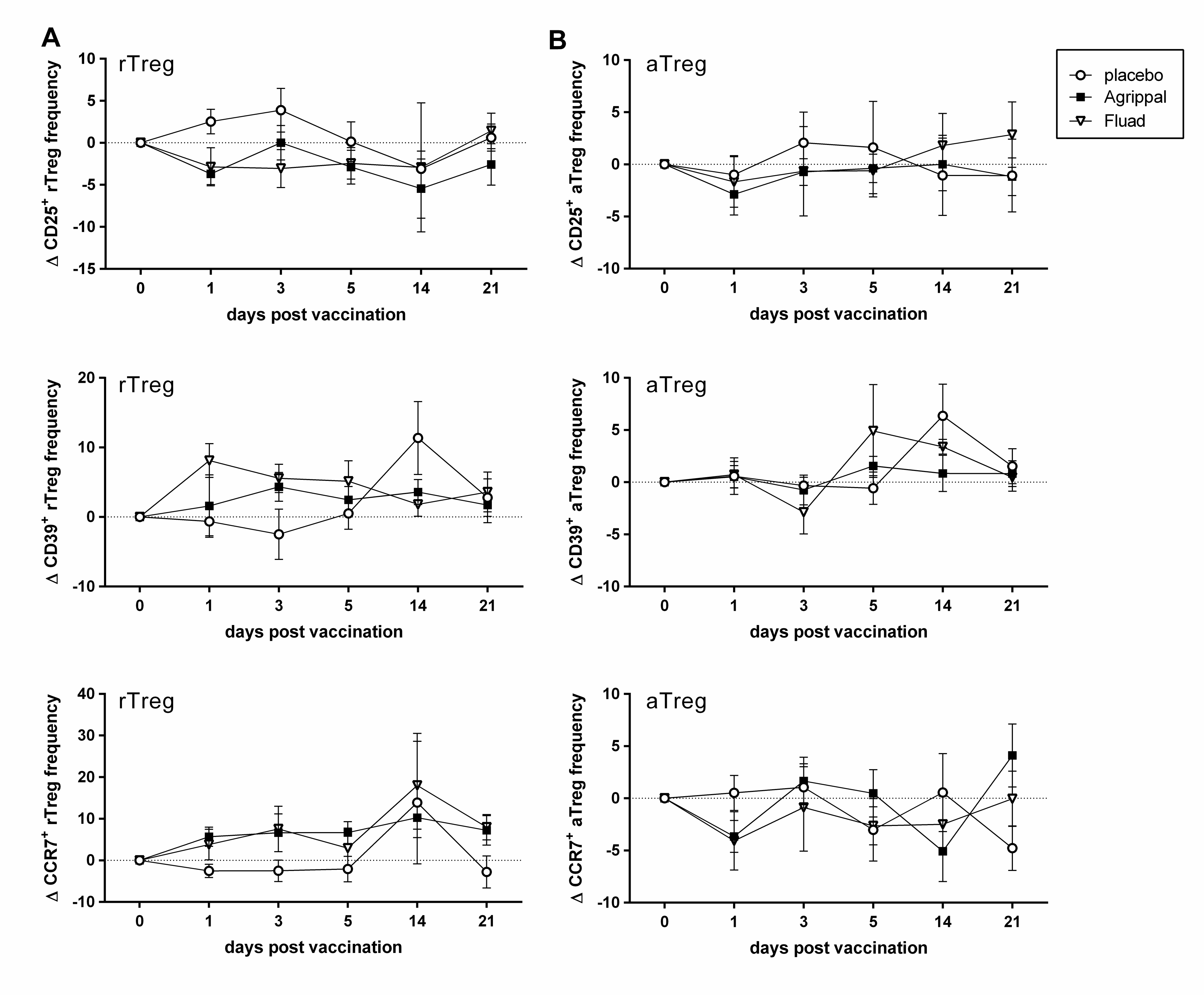

Supplement: S2 Fig — At day 0, healthy adults were i.m. vaccinated with a non-adjuvanted (Agrippal®) or MF59®-adjuvanted (Fluad®) trivalent influenza subunit vaccine or injected with a placebo. At day 1, 3, 5, 14 and 21 post vaccination changes in Treg frequency and phenotype were determined. The delta Treg percentage per time point was determined per donor (= % Treg dayx—% Treg day0). (A) Mean (± SEM) delta percentage of CD25, CD39 and CCR7 expression on rTreg after vaccination (top to bottom). (B) Mean (± SEM) delta percentage of CD25, CD39 and CCR7 expression on aTreg after vaccination (top to bottom). Means were statistically compared with a Kruskal-Wallis test. (TIF) [file pone.0179942.s002.tif]

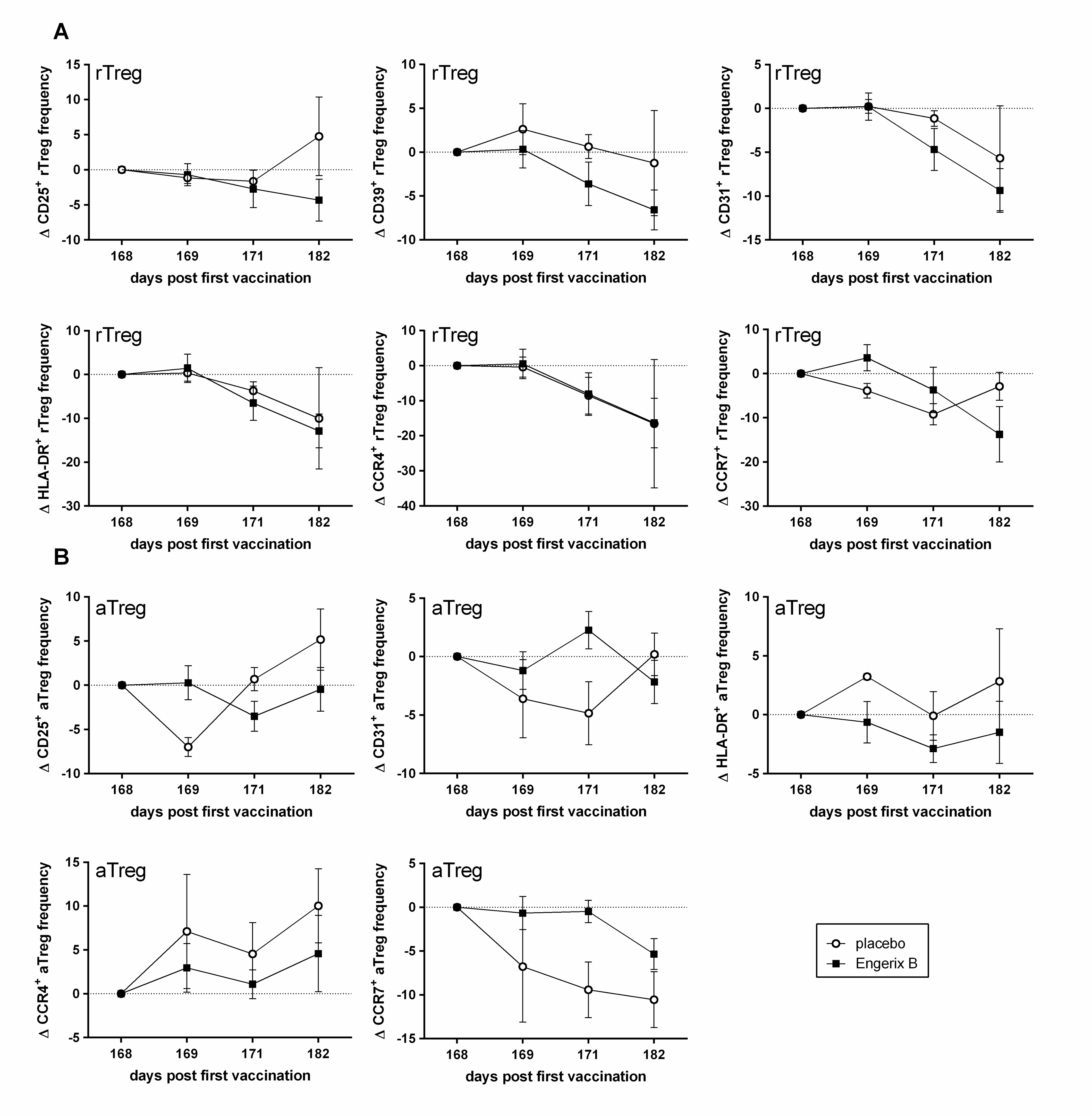

Supplement: S3 Fig — At day 0, day 28 and day 168, healthy adults were i.m. vaccinated with Engerix-B® or injected with a placebo. At day 1, 3, and 14 after the third immunization dose (day 169, 171 and 182) changes in Treg frequency and phenotype were determined. The delta Treg percentage per time point was determined per donor (= % Treg dayx—% Treg day168). (A) Mean (± SEM) delta percentage of CD25, CD39, CD31, HLA-DR, CCR4 and CCR7 expression on rTreg after vaccination. (B) Mean (± SEM) delta percentage of CD25, CD31, HLA-DR, CCR4 and CCR7 expression on aTreg after vaccination. Means were statistically compared with an upaired, two-tailed Mann-Whitney test. (TIF) [file pone.0179942.s003.tif]

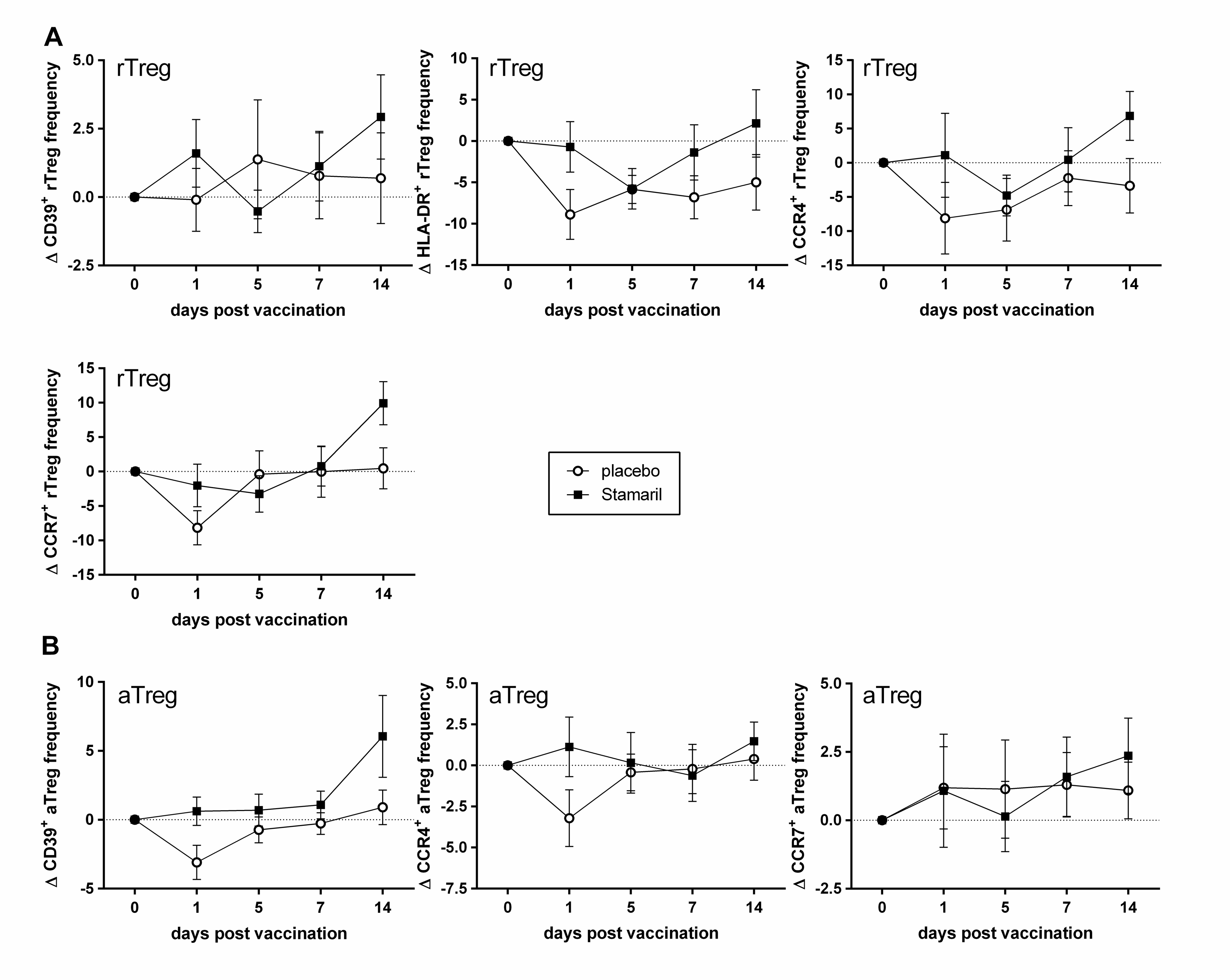

Supplement: S4 Fig — At day 0, healthy adults were s.c. vaccinated with Stamaril® or injected with a placebo. At day 1, 5, 7 and 14 post vaccination changes in Treg frequency and phenotype were determined. The delta Treg percentage per time point was determined per donor (= % Treg dayx—% Treg day0). (A) Mean (± SEM) delta percentage of CD39, HLA-DR, CCR4 and CCR7 expression on rTreg after vaccination. (B) Mean (± SEM) delta percentage of CD39, CCR4 and CCR7 expression on aTreg after vaccination. Means were statistically compared with an unpaired, two-tailed Mann-Whitney test. (TIF) [file pone.0179942.s004.tif]

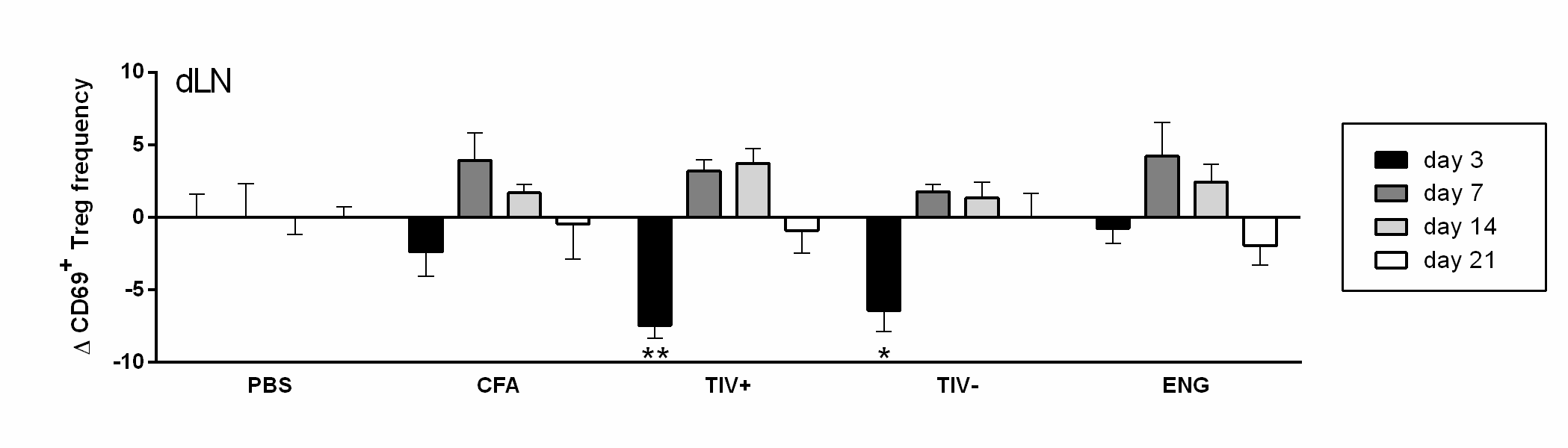

Supplement: S5 Fig — At day 0, mice were i.m. injected with CFA or with one of the vaccines (TIV+, TIV- and Engerix-B®). Mock-injected animals received PBS. At day 3, 7, 14 and 21 post vaccination changes in CD69 expression on Treg were determined in the dLN. The delta CD69 percentage per time point was determined by comparing the treatment with the mean of PBS (= % CD69 treatment dayx—average %CD69 PBS dayx). The mean percentage CD69 in PBS-injected mice was 27.6 ± 4.5%. Mean (± SEM) delta percentage are indicated. N = 3–7 mice per group. Differences were determined with a one-way ANOVA followed by Dunnett’s multiple comparisons test (* p < 0.05, ** p < 0.01 relative to placebo at the same time point). TIV+: TIV supplemented with MF59® adjuvant; TIV-: TIV only; ENG: Engerix-B®. (TIF) [file pone.0179942.s005.tif]

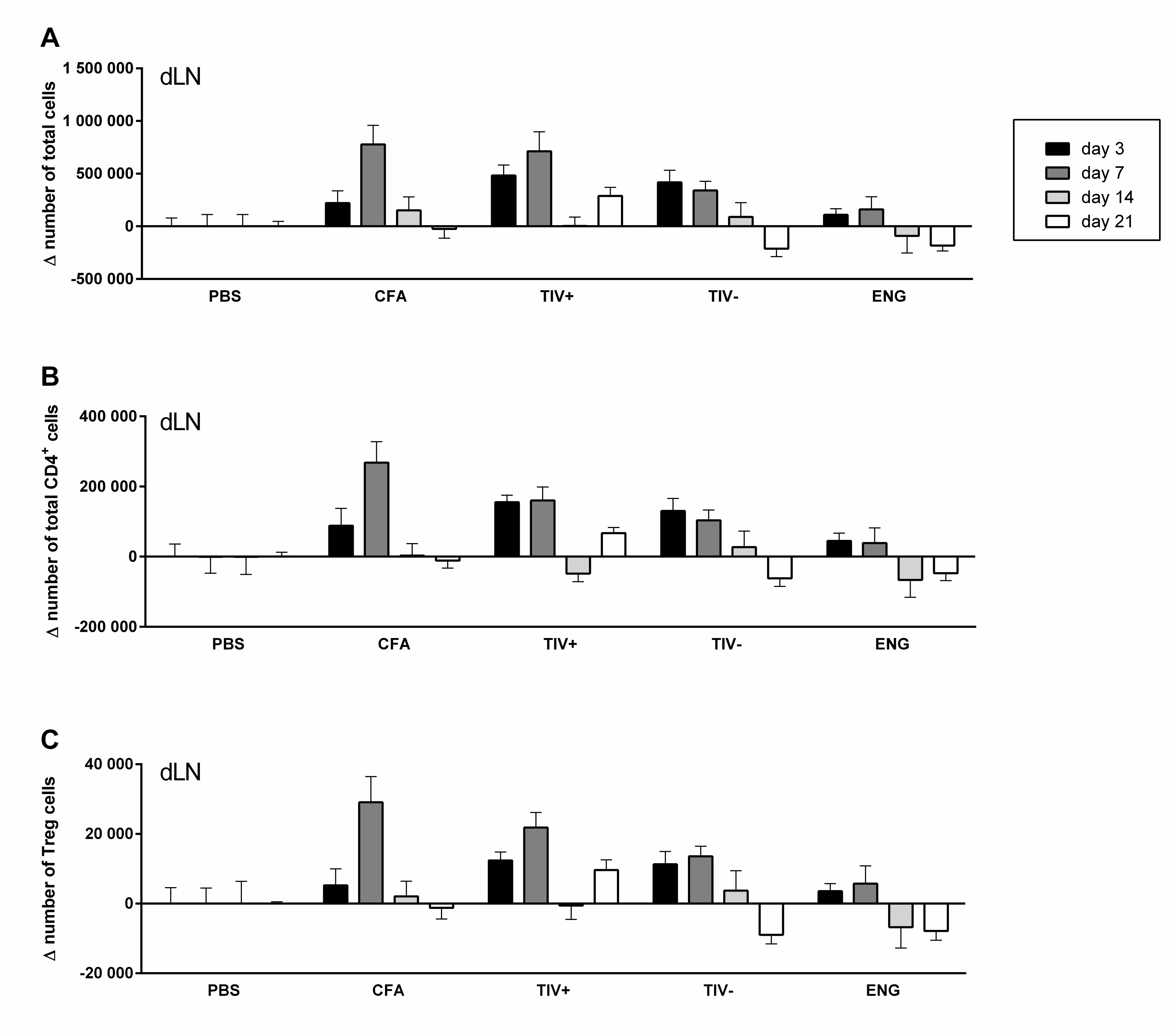

Supplement: S6 Fig — At day 0, mice were i.m. injected with LPS, CFA or with one of the vaccines (TIV+, TIV- and Engerix-B®). Mock-injected animals received PBS. At day 3, 7, 14 and 21 post vaccination changes in numbers of (A) total cells, (B) CD4+ T cells and (C) Treg in the dLN were determined. The delta value per time point was determined by comparing the treatment with the mean of PBS (= number of cells (treatment) dayx—average number of cells (PBS) dayx). The mean number of cells in PBS-injected mice was 7.3x10^5^ ± 3.7x10^5^ (total cell numbers), 3.0x10^5^ ± 1.6x10^5^ (CD4+ T cells) and 3.3x10^5^ ± 1.9x10^5^ (Treg). Mean (± SEM) delta cell numbers are indicated. N = 3–7 mice per group. TIV+: TIV supplemented with MF59® adjuvant; TIV-: TIV only; ENG: Engerix-B®. (TIF) [file pone.0179942.s006.tif]
