# Supplementary material for: Regulatory T cell frequencies and phenotypes following anti-viral vaccination
Source: PLoS One. 2017 Jun 28;12(6):e0179942. doi: 10.1371/journal.pone.0179942 (PMC5489208; doi:10.1371/journal.pone.0179942)
Supplement: S2 Text — (PDF) [file pone.0179942.s008.pdf]

A clinical study to generate an exploratory training set of data characterising clinical events, physiological and metabolic responses, and innate and adaptive immune responses following the first and third of three intra-muscular immunisations with "Engerix B" Hepatitis B vaccine or saline placebo in healthy adults with no pre-existing immunity to Hepatitis B

Protocol Number: **CRC305B**

Version Number: **Final 6.0**

Date: **05 December 2013**

Confidentiality Statement

The information contained in this document is the property of the University of Surrey Clinical Research Centre (Surrey CRC) and is provided to you in confidence as an investigator, potential investigator, sponsor, or consultant, for review by you, your staff and an applicable Ethics Committee (EC). It is understood that this information will not be disclosed to others without written authorisation from the Surrey CRC, except to the extent necessary to obtain written informed consent from those persons to whom the drug may be administered.

1 CLINICAL STUDY PROTOCOL AGREEMENT FORM

Authorisation of final version

A clinical study to generate an exploratory training set of data characterising clinical events, physiological and metabolic responses, and innate and adaptive immune responses following the first and third of three intra-muscular immunisations with "Engerix B" Hepatitis B vaccine or saline placebo in healthy adults with no pre-existing immunity to Hepatitis B

On behalf of the Sponsor (University of Surrey):

Job Title

Signature ……………………………. Date ……………………….
Name:

Principal Investigator Agreement

I agree to conduct this study according to this protocol and to comply with its requirements, subject to ethical principles that have their origins in the Declaration of Helsinki, safety considerations and the applicable laws and regulations, I agree to conduct the study in accordance with local regulations and the International Conference on Harmonisation Topic E6: Guideline for Good Clinical Practice (ICH GCP).

I will promptly submit the protocol to applicable ethical review board(s). I agree not to make any changes to the protocol without agreement from the sponsor and prior review and written approval from the local Ethics Committee, except where necessary to halt an immediate threat to subject safety, or for administrative study details when such actions are permitted by local regulations.

I understand fully the appropriate use of "Engerix B" as described in this protocol, and any other relevant material made available by the sponsor.

I will make certain that all personnel assisting with the study will be adequately informed about the study medication and their study-related duties as described in the protocol.

I understand that, should the decision be made by the Sponsor to terminate prematurely or suspend the study, at any time and for whatever reason, such decision will be communicated to me in writing. Conversely, should I decide to withdraw from execution of the study, I will communicate immediately in writing to the Sponsor or their representatives.

Principal Investigator

Signature ……………………………. Date ……………………….

Name:

2 TABLE OF CONTENTS

1 CLINICAL STUDY PROTOCOL AGREEMENT FORM 2
2 TABLE OF CONTENTS 3
3 LIST OF ABBREVIATIONS AND DEFINITIONS 6
4 CONTACT INFORMATION 6
5 PROTOCOL SYNOPSIS 9
6 STUDY FLOW CHARTS 13
6.1 Study Flow Chart 13
6.2 Visit 2 Inpatient Flow Chart 14
7 SUMMARY OF STUDY DESIGN AND RATIONALE 15
7.1 Introduction and Study Rationale 15
7.2 Summary of Study Design 19
7.3 Profile of Study Drugs 19
7.4 Potential Risks and Benefits 20
7.5 Rationale For Study Design 20
7.5.1 Rationale for doses and schedule 20
7.5.2 Rationale for Study Endpoints 20
7.5.1 Blinding 21
8 STUDY OBJECTIVES AND ENDPOINTS 21
8.1 Exploratory Study Objectives 21
8.2 Study Endpoints (study variables measured) 22
9 SUBJECT SELECTION AND WITHDRAWAL CRITERIA 23
9.1 Population base 23
9.2 Inclusion criteria 23
9.3 Exclusion criteria 24
9.4 Discontinuation and Withdrawal of subjects from the study 25
10 CLINICAL STUDY PROTOCOL 26
10.1 Study Procedures 26
10.2 Duration of Study Participation 27
10.3 Study Restrictions 27
10.3.1 Dietary Restrictions 27
10.3.2 Activity Restrictions 27
10.3.3 Alcohol Restrictions 27
10.3.4 Caffeine Restrictions 28
10.3.5 Smoking Restrictions 28
10.4 Prohibited Medications and Non-Drug Therapies 28
11 STUDY MEDICATION 29
11.1 Treatment Plan 29
11.2 Preparation, Administration and Dosage of Study Medication 29
11.2.1 Preparation Instructions 29
11.2.2 Route of administration 29
11.2.3 Dose and Dosing Schedule 29
11.3 Packaging and Labelling 29
11.4 Storage and Accountability of Drug Supplies 29
11.5 Return of Clinical Supplies 29
11.6 Responsibilities 30

11.7 Compliance ... 30
11.8 Treatment Assignment Procedures ... 30
11.8.1 Randomisation ... 30
11.8.2 Blinding/Unblinding ... 30
12 STUDY DATA PARAMETERS ... 31
12.1 Laboratory Assessments ... 31
12.1.1 Study Blood Volume ... 31
12.1.2 Urinalysis ... 33
12.2 Vital signs ... 33
12.3 Measurement of temperature & injection site reactions ... 33
12.4 Methods and Timing for Assessing and Recording Study Data Parameters ... 33
13 SAFETY MONITORING ... 33
13.1 Specification of Safety Parameters ... 33
13.1.1 Laboratory and Other Safety Assessment Abnormalities Reported as AEs and SAEs ... 33
13.1.2 Pregnancy ... 34
13.1.3 Definition of an AE ... 34
13.1.4 Definition of a SAE ... 35
13.2 Evaluating and Recording Adverse Events ... 35
13.3 Intensity of Event ... 35
13.4 Relationship to study product ... 37
13.5 Expectedness of SAEs ... 38
13.6 Reporting of Serious Adverse Events ... 38
13.7 Progress reporting ... 39
13.8 Treatment and Follow-up of Adverse Events ... 40
13.9 Overdose of Study Medication ... 40
13.9.1 Management of Overdose ... 40
14 STATISTICAL CONSIDERATIONS AND ANALYTICAL PLAN ... 41
14.1 Responsibility for Analysis ... 41
14.2 Justification of Sample Size ... 41
14.3 Definition of Study Completion ... 41
14.4 Definition of Criteria for Termination of the Study ... 41
14.5 Analysis of Clinical Events and Exploratory Endpoints after immunisation ... 42
15 STUDY DOCUMENTATION ADMINISTRATION ... 42
15.1 Source documents and CRF/ eCRF ... 42
15.2 Monitoring ... 43
15.3 Access to Source Data Documents ... 43
15.4 Data Handling and Record Retention ... 43
15.5 Subject Confidentiality and Data Protection ... 43
16 QUALITY CONTROL AND QUALITY ASSURANCE ... 44
17 CLINICAL STUDY PROTOCOL DEVIATIONS AND AMENDMENTS ... 44
18 CONDITIONS FOR TERMINATING THE STUDY ... 44
19 ETHICAL AND REGULATORY REQUIREMENTS ... 45
19.1 Informed Consent ... 45
19.2 Independent Ethics Committee (IEC) ... 45
19.3 Annual/Final Reports ... 46
20 FINANCE AND INSURANCE ... 46

21 PUBLICATIONS 46
22 REFERENCES 46

3 LIST OF ABBREVIATIONS AND DEFINITIONS

Example Text:

AE	Adverse Event
BP	Blood Pressure
CRF	Case Report Form
EU	European Union
GCP	Good Clinical Practice
GP	General Practitioner
HBV sAb, sAg, cAb	Hepatitis B Virus surface antibody/antigen, core antibody
ICH	International Conference on Harmonisation
IEC	Independent Ethics Committee
MHQ	Medical History Questionnaire
NMC	Nursing and Midwifery Council
SAE	Serious Adverse Event
SmPC	Summary of Product Characteristics
SOPs	Standard Operating Procedures
Surrey CRC	Surrey Clinical Research Centre
UK	United Kingdom

4 CONTACT INFORMATION

PRINCIPAL INVESTIGATOR

Name: Professor David Lewis
Address: Surrey Clinical Research Centre, Egerton Road, Guildford, GU2 7XH
Tel: 01483 689797
Fax: 01483 689790
Email: d.j.lewis@surrey.ac.uk

Principal Study Physician

Name: Dr Aldona Greenwood
Address: Surrey Clinical Research Centre, Egerton Road, Guildford, GU2 7XH
Tel: 01483 683442
Fax: 01483 689790
Email: a.greenwood@surrey.ac.uk

COLLABORATING LABORATORIES

Partnership Pathology Services (PPS)
Royal Surrey County Hospital
Egerton Road
Guildford
Surrey GU2 7XX
Main contact: Andrew Mason, Lyn Hurst, Sue Mackie
Tel. 01483 571122
Email: andrew.mason1@nhs.net; lynhurst@nhs.net, sue.mackie@fph-tr.nhs.uk

Department of Medical Microbiology
West Park Hospital
Horton Lane
Epsom, Surrey, KT19 8PB
Main contact: Sharon Chambers
Email: Sharon.Chambers@esth.nhs.uk

Max Planck Institute for
Infection Biology (MPIIB)
Department for Immunology
Chariteplatz 1
10117 Berlin
Germany
Contact person: Dr Gayle McEwen
Email: mcewen@mpiib-berlin.mpg.de

Chalmers University of Technology
SE-412 96 Gothenburg
Sweden
Head of Lab: Intawat Nookaew
Email: intawat@chalmers.se

GlaxoSmithKline Vaccines
Building RIX 43N206
Rue de L'institut 89
1330 Rixensart
Belgium
Contact: Dr Philippe Denoel
Email: philippedenoel@gskbio.com

University of Surrey
Leggett Building
Daphne Jackson Road, Manor Park
University of Surrey
Guildford, Surrey, GU2 7WG
Contact: Professor David Lewis
Tel. 01483 68 8306
Email: D.J.Lewis@surrey.ac.uk.

Commissariat a L'Energie Atomique et aux Energies Alternatives (CEA)
Division of Immuno-Virology
Route du Panorama
Fontenay aux Roses
92265
France
Contact: Professor Roger Le Grand
Tel. 0033 1465 48757
Email: roger.le-grand@cea.fr

deCODE genetics
Sturlugata 8, Reykjavik
IS-101 Iceland
Contact: Ingileif Jónsdóttir
Email: Ingileif.Jonsdottir@decode.is

Other laboratories at Biovacsafe collaborators may be used for the analysis of other laboratory parameters.

5 PROTOCOL SYNOPSIS

**Title**	**A clinical study to generate an exploratory training set of data characterising clinical events, physiological and metabolic responses, and innate and adaptive immune responses following the first and third of three intra-muscular immunisations with "Engerix B" Hepatitis B vaccine or saline placebo in healthy adults with no pre-existing immunity to Hepatitis B**
**Sponsor**	University of Surrey
**Principal Investigator**<br>**Study Location**	David JM Lewis, MD<br>Surrey Clinical Research Centre, University of Surrey
**Indication**	Biomarkers of vaccine safety and immunogenicity
**Exploratory Study Objectives**	To generate an **exploratory training set of data**, to undergo integrated systems biology analysis to identify putative biomarkers of responses to immunisation, for validation in subsequent studies or in animal models.<br><br>The training data set will include data characterising:<br>1. Physiological responses at various time points after immunisation by measuring:<br>a. Local and systemic vaccine-related clinical events.<br>b. Physiological assessments: heart rate, temperature, blood pressure.<br>c. Haematology (blood counts and ESR), Biochemistry (liver, renal and bone panels) parameters.<br>2. Metabolic, innate and adaptive immune responses including:<br>a. Innate immune activation detected by global gene expression in whole blood<br>b. Metabolism detected by metabolite concentrations in serum<br>c. Adaptive immunity determined by serum anti-hepatitis B IgG titre<br>d. Innate and adaptive immune activation detected by gene pathway activation in whole blood<br>e. Metabolism detected by metabolic gene expression and pathway activation in whole blood<br>f. Immune activation detected by:<br>i. Concentration of selected inflammatory soluble mediators in serum including:

	1. chemokines and cytokines<br>2. acute phase proteins<br>ii. PBMC cytokine secretion, proliferation or surface markers in response to *in vitro* antigen stimulation<br>3. Genotype of subjects<br>4. Correlations in changes in innate immune activation and metabolism with adverse events, haematology and biochemistry panels, genotype and physiological assessments
**Study Design**	Partial-blind (subject and laboratory blinded), randomised, placebo controlled exploratory "training study".
**Population**	24 Healthy adults
**Main Selection Criteria**	• Age: 18 – 45 years<br>• Male: Female<br>• Healthy: no active disease process that could interfere with endpoints measured as determined by medical history<br>• Not taking regular medications that could interfere with endpoints measured<br>• Seronegative to Hepatitis B sAb, sAg and cAb at enrolment<br>• Never received Hepatitis B vaccine or contracted hepatitis B infection<br>• No contraindications to "Engerix B" vaccine
**Study Medicinal Products:**<br>Formulations<br>Route of Administration<br>Dose regimen	• "Engerix B" Hepatitis B vaccine<br>• Three 1 mL doses administered at 0, 1 and 6 months<br>• Intra-muscular<br>• 20 subjects
**Concurrent Controls**	• Saline placebo 1 mL<br>• Intra-muscular<br>• Three 1 mL doses administered at 0, 1 and 6 months<br>• 4 subjects

**Exploratory Study Endpoints (study variables measured)**	1. Frequency of local and systemic vaccine-related clinical events at **all** time points from vaccination up to last study visit.<br>2. Change from pre-immunisation baseline values in heart rate, temperature, blood pressure at **all** time points from time of immunisation up to last study visit.<br>3. Change from pre-immunisation baseline values in haematology (blood counts and ESR), biochemistry (liver, renal and bone panels) parameters at **selected** time points from time of immunisation up to last study visit.<br>4. Change from pre-immunisation baseline values in global gene expression measured on whole blood samples at **selected** time points from time of immunisation up to last study visit<br>5. Change from pre-immunisation baseline values in metabolite concentrations in serum samples at **selected** time points from time of immunisation up to last study visit<br>6. Change from pre-immunisation baseline values in serum anti-hepatitis B IgG titre in serum samples at **selected** time points from time of immunisation up to last study visit<br>7. Change from pre-immunisation baseline values in metabolic gene expression and pathway activation measured on whole blood samples at **selected** time points from time of immunisation up to last study visit<br>8. Change from pre-immunisation baseline values in concentration of selected cytokines and acute phase proteins in serum samples at **selected** time points from time of immunisation up to last study visit<br>9. Change from pre-immunisation baseline values in PBMC cytokine secretion, proliferation or surface markers in response to *in vitro* antigen stimulation at **selected** time points from time of immunisation up to last study visit<br>10. Fold increase in serum hepatitis B IgG titre at **selected** time points after immunisation compared with pre-immunisation baseline.<br>11. Genotype of subject
**Assessment Schedule**	**Visit 1 – Screening Visit (Day – 28 to -2)**<br>• Informed Consent<br>• Demography<br>• Medical history and concomitant medication<br>• Vital signs (blood pressure, heart rate, oral temperature)<br>• Assessment of inclusion/exclusion criteria

	• Height, weight, BMI<br>• Clinical samples as per Table 1<br>• Blood pregnancy test<br>• Physical examination<br><br>**Visits 2 and 7 – Residential Visit with Immunisation (Days -1 to +5 after doses #1 and #3)**<br>• 7 day/6 night inpatient stay<br>• Continued eligibility check<br>• Concomitant medications<br>• Clinical samples as per Table 2<br>• Randomisation (visit 2)<br>• Vaccine administration<br>• AEs recorded<br>• Vital signs (blood pressure, heart rate, oral temperature)<br>• Blood sample for DNA genotype<br><br>**Visits 3, 4, 5 & 6; 8, 9, 10 &11 – Follow Up Visits (Days +7, +14, +21 & +28 after doses #1 and #3)**<br>• Vital signs (blood pressure, heart rate, temperature)<br>• Continued eligibility check<br>• Concomitant medications<br>• Clinical samples as per Table 1<br>• Blood pregnancy test (Final Visit only)
**Data Analysis**	The Surrey CRC Statistics Team will list Serious AEs by study visit, by severity, by relationship to study drug, by System Organ Class and Preferred Term, and by study group. Non-serious AEs will be listed separately.<br>Exploratory endpoints will be analysed by a Systems Biology approach to identify exploratory biomarkers for confirmation in subsequent validation trials.
**Duration of Study Period (per subject)**	7 months

6 STUDY FLOW CHARTS

6.1 Study Flow Chart

Table 1: Study overview flowchart

Procedures	Screening	I/P stay #1	F/U	F/U	F/U	F/U	I/P stay #2	F/U	F/U	F/U	F/U
Visit number	1	2	3	4	5	6	7	8	9	10	11
Day number	-28 to -2	-1 to +5	7	14	21	28	168 to 173	175	182	189	196
Month number		0				1	6				7
Prime/boost cycle		Cycle 1: Prime					Cycle 2: Boost				
Written informed consent	X										
Demographic data	X										
Medical history	X										
Physical exam	X										
Vital signs [a]	X	See table 2	X	X	X	X	See table 2	X	X	X	X
Laboratory safety [b]	X	See table 2	X	X	X	X	See table 2	X	X	X	X
Screening serology (hepatitis C, HIV)	X										
Pregnancy Test: Blood / Urine [c]	B	See table 2									B
Blood for DNA genotype		X									
Hepatitis B serology for eligibility	X										
Vaccine administration		See table 2				X	See table 2				
Whole blood for gene expression (RNA)		See table 2	X	X	X	X	See table 2	X	X	X	X
Serum immunology		See table 2	X	X	X	X	See table 2	X	X	X	X
Acute Phase proteins		See table 2	X	X	X	X	See table 2	X	X	X	X
Metabolomics		See table 2	X	X	X	X	See table 2	X	X	X	X
Chemokines & Cytokines		See table 2	X	X	X	X	See table 2	X	X	X	X
Biobank		See table 2	X	X	X	X	See table 2	X	X	X	X
PBMCs for immunology		See table 2	X	X	X	X	See table 2	X	X	X	X
Recording of adverse events after immunisation		Continuous	--	--	--	--	Continuous	--	--	--	--

[a] Heart rate, blood pressure, oral temperature
[b] FBC, Electrolytes, Renal & Liver panels, Ca/Phosph panel, ESR, urinalysis, glucose.
[c] Female subjects only

6.2 Visit 2 Inpatient Flow Chart

Table 2: Inpatient Schedule

Inpatient Day	#1				#2					#3						#4						#5						#6						#7				
Days after vaccine #1 or #3	-1				0					+1						+2						+3						+4						+5				
Time of day[8]	*08*	*12*	*16*	*24*	*08*	*12*	*16*	*20*	*24*	*04*	*08*	*12*	*16*	*20*	*24*	*04*	*08*	*12*	*16*	*20*	*24*	*04*	*08*	*12*	*16*	*20*	*24*	*04*	*08*	*12*	*16*	*20*	*24*	*04*	*08*	*12*	*16*	*20*
Subject arrival	X																																					
Subject free to depart																																						x[a]
Pregnancy Test: Blood / Urine	U																																					
Vaccine or placebo administration					X																																	
Vital Signs [b]	x	x	x	x	x	x	x	x	x	x	x	x	x	x	x	x	x	x	x	x	x	x	x	x	x	x	x	x	x	x	x	x	x	x	x	x	x	x
Laboratory safety [c]	x				x						x						x						x						x						x			
Blood for DNA genotype	X																																					
Whole blood for gene expression (RNA)	x	x	x	x	x	x	x	x	x	x	x	x	x	x	x	x	x	x	x	x			x			x			x			x			x			x
Acute phase proteins	x				x						x						x						x						x						x			
Metabolomics & Biobank	x	x	x	x	x	x	x	x	x		x			x			x			x			x			x			x			x			x			x
Chemokines & Cytokines	x				x						x			x			x						x						x						x			
Serum Immunology	x																																					
PBMCs for immunology					x						x												x												x			
Adverse events [d]	<— >																																					

*Representative times for one subject. Dosing times will be staggered between subjects and all procedures conducted relative to vaccine or placebo administration

[a] Subjects can depart at 20:00 on inpatient day #7 or stay overnight and depart next day. Subject returns day +7 as shown in Table 1.

[b] As table 1

[c] As Table 1.

[d] Includes evaluation of injection site (pain, redness, swelling, induration, allergic rashes)

7 SUMMARY OF STUDY DESIGN AND RATIONALE

7.1 Introduction and Study Rationale

Currently licensed vaccines are widely accepted to be safe and to have an acceptable reactogenicity profile. Vaccine development lead-times are extremely long and expensive due to the requirements for the extensive safety and efficacy testing required prior to Market Authorization. Rarely, significant adverse reactions have been detected post-licensure that were not detected during the development of the vaccine and only became apparent during large-scale Phase IV post marketing surveillance (e.g. intussusception, Bell's palsy). Such events have led to the withdrawal of vaccines from the market, resulting in financial impact, loss of confidence in vaccines, and individual harm to those affected and potentially to those exposed to vaccine-preventable diseases by decreases in immunization rates.
Clinical trials during the early stage pre-marketing assessment of safety and efficacy are not designed or powered to study immunopathological responses intensively enough to detect *transient* or *infrequent* phenomena (e.g. auto-reactive B and T cells, inflammation), or in sufficient *depth* (frequency of sampling, application of advanced technology) to be able to predict rare or subtle events which, in susceptible recipients, may subsequently trigger or lead to exacerbation of autoimmune or inflammatory diseases.

Vaccines are thought to trigger innate inflammatory responses to induce antigen-specific adaptive immunity (the desired effect of a vaccine), but excessive inflammation may lead to serious inflammatory complications or unwanted side effects. A lack of reliable biomarkers predicting severe inflammation has halted several exploratory vaccines, and withdrawn some licensed vaccines, some of which were associated with inflammatory complications, albeit low frequency, in some individuals. The BIOVACSAFE project, a 5-year €30M project funded by the Innovative Medicine Initiative, will undertake a **series of correlated clinical studies** that will apply and develop technologies to generate *clinical* data on inflammation with licensed vaccines as benchmarks, and identify biomarkers to predict acceptable reactogenicity, for correlation with standardized clinical readouts and inflammatory markers assessed in natural infections.

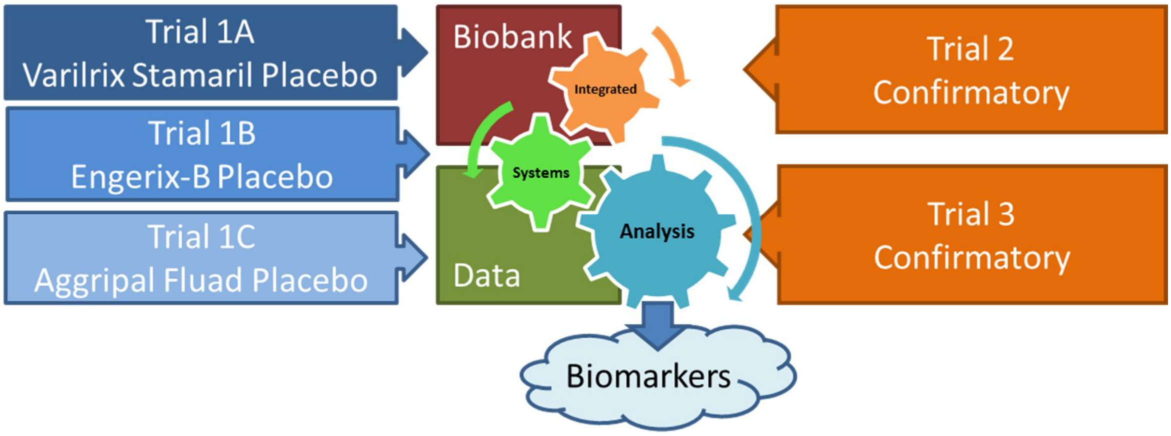

The overall project will follow the general structure of three studies employing five different vaccines plus placebo groups that will generate a biobank of samples and a

set of data (clinical responses, gene responses, proteins and immunology responses). Apart from using different vaccines, each study will follow the same protocol to allow the data to be combined into one dataset and biobank. Integrated systems biology analysis of the data and iterative access to the biobank, will identify putative "biomarkers" of inflammation. Two larger clinical trials will then follow which will confirm or refute the validity of these putative biomarkers. These two trials will also add to the database and biobank to allow further rounds of integrated systems biology analysis to generate hypotheses and putative biomarkers for future research projects. The scheme is illustrated in the diagram above.

The combination of studying basic responses at the protein, gene and metabolic level is often termed "biomics". Biomics can be exploited to discover ***biomarkers*** (a measurable biological response that predicts something), which may be useful for monitoring of vaccine trials, and ideally can predict occurrence of beneficial and detrimental effects that are directly correlated with side effects and efficacy of vaccines undergoing clinical testing in humans. The different biomics include transcriptomics (genes), metabolomics (lipids and metabolites) and proteomics (proteins). In this project transcriptomics and metabolomics, together with profiling of cytokines and chemokines (molecules produced in immune responses) will be harnessed for assessment of vaccine responses with an emphasis on immunosafety and immunogenicity. Global gene expression profiling by transcriptomics has led to the definition of biosignatures, which can be used to discriminate diseased from healthy individuals. More recently, such studies have also been exploited for monitoring of vaccine effects in vaccinees (Pulendran et al., 2010).

Blood transcriptional profiles reflect the immune status of the host and expression patterns change upon infection and early inflammation. Cytokine profiles are linked to the blood transcriptome because they are, in part, influenced by leukocyte gene expression, but contain additional information resulting from processes occurring in extra-vascular tissues. Metabolomics identifies and quantifies small molecules to provide insights into changes in general metabolomic processes, while multiplex cytokine/chemokine profiling provides a "batch immune answer" of inflammatory processes. Both of these platforms complement transcriptomics and are known to be good predictors for disease and/or inflammation, underlining their relevance to vaccine safety. The technologies used in this clinical study are integrated with other technologies to deliver state of the art biomics and systems biology analysis for an integrated set of Clinical Studies (parallel studies using the same vaccines in animal models), and related population-based studies of infections and allergy. The clinical studies of which this is one will closely integrate with other activities within the BIOVACSAFE project to translate biomarker discovery into practical tools for vaccine development and regulation.

The characterization of early innate immune system events following immunization (days 0-3, 7-30) by gene expression and multiplex cytokine / cellular response analysis (systems biology approach) have been used to successfully identify biomarkers of inflammation and immune response/efficacy. This has been done in a predictive and reproducible way by immunizing relatively small numbers of healthy subjects, for example with live viral Yellow Fever vaccine (Querec et al., 2009) or adjuvanted subunit malaria vaccines (Vahey et al., 2010) This opens-up the novel possibility to conduct intensive and highly focused, but relatively small clinical trials

early-on in the development of novel vaccine technologies to identify biomarkers that may be predictive of safety signals that may only become apparent in subsequent larger scale clinical testing or during post marketing surveillance.

We propose to apply a systems biology approach to identify predictive biomarkers of vaccine immunosafety in the context of an initial “Training Study” that incorporate intensive clinical monitoring of vaccine recipients, standardized Adverse Event definitions, accepted measurements of immune responses and in which the analytic plan will strive to correlate biomarker activity with observed physiological and immunological responses to vaccination. In addition, while studies of transcriptomics or other biomics technologies have been used in previous studies, we will for the first time bring together metabolomics, transcriptomics and other biomics technologies, together with readouts of immune-efficacy and standardized definitions of adverse reactions, to the simultaneous evaluation relevant licensed vaccines studied under a highly standardized clinical setting to assess the relationships between these biomarkers and the short term reactogenicity of, and immune responses to, the selected vaccines. We will, in particular, use detailed metabolic models to link transcription data with metabolomics data and hereby potentially improve the statistical power in terms of biomarker identification. If successful, these biomarkers could be used in early stage clinical trials to optimize selection of vaccine candidates with a profile that will be unlikely to generate worrisome safety signals once they are in generalized use.

Following this intense series of studies in small numbers of subjects, a very large dataset of clinical, and biological data will be generated together with a biobank of samples. Integrated systems biology analysis will then be used where the biobank can be accessed to study relevant time points, a model of responses built with biomarkers associated with clinical events identified, and then the biobank re-accessed to further extend the analysis in an iterative way. Based on the preliminary findings we will then design the second and third large outpatient clinical trials to confirm the results of the systems biology analysis. This is illustrated in the figure:

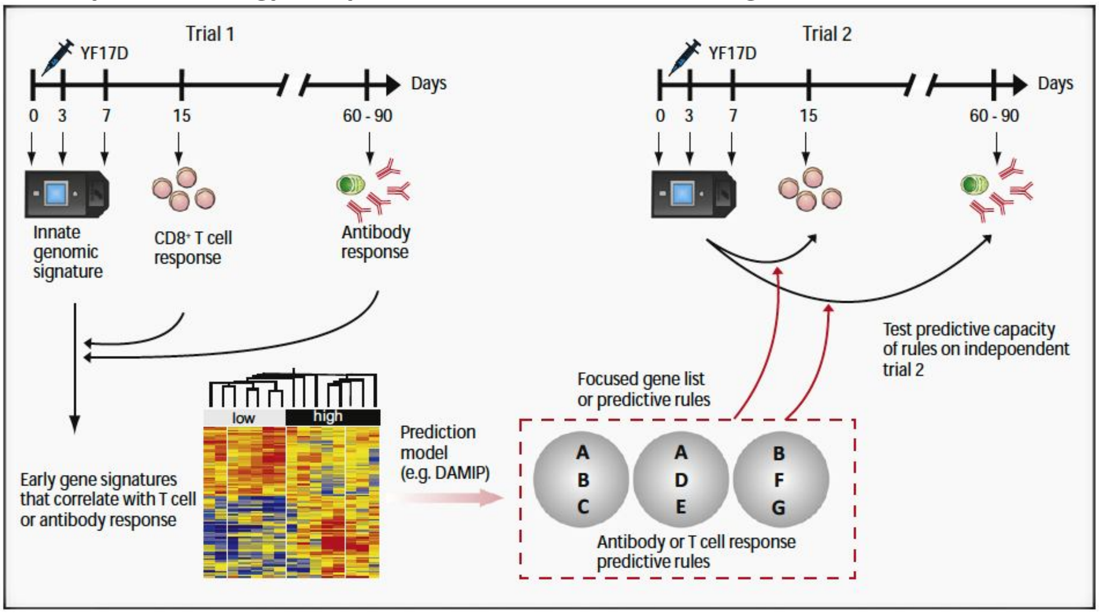

An important feature of the BIOVACSAFE project is the fact that we will restrict to the study of licensed vaccines which are accepted by regulatory authorities as being safe, well tolerated, and sufficiently efficacious to warrant their recommendation for general use. We do not have access to vaccines which have been shown in pre- or post-marketing studies to be unsafe or non-efficacious. Thus, the conclusions that can be drawn from these studies must be understood in the context of the null hypothesis that vaccines having the biomarker profiles identified in these studies do not present a potential safety risk for the general population. This does not mean that in certain genetically predisposed individuals, or in persons with active underlying autoimmune disease, these vaccines could potentially trigger or exacerbate autoimmune disease.

In the first intense training study group size will be 20 subjects randomized per vaccine treatment group, we will attempt to provide broadly equal male to female ratios per group where possible. This sample size is sufficient for transcriptomics and metabolomics analysis under the provision of closely matched subjects and robust biomarkers, and the ability to evaluate the whole closely-matched subjects from the combined studies in the series to identify background. A sample size of 20 per treatment group is also thought to be sufficient for a training study as each of the licensed vaccines to be tested is known to have a high (>80%) rate of vaccine “take” in healthy adult populations.

Subjects in the placebo groups of each of the studies within this coordinated study will be pooled to give a placebo group size of at least 20. Consideration will be given in the analysis to the different routes of immunisation (SC and IM). In addition, each subject acts as their own control for kinetics with comparison from the baseline pre-immunisation levels of biomarkers measured.

Due to the large numbers of individual samples generated across the proposed time course of monitoring, and restrictions on budget, we will biobank all samples at all time points measured. Subsequently, in collaboration with parallel studies in animal models and based on the observed clinical signs and symptoms for each vaccine, analysis of selected time points will be undertaken for all subjects to identify biomarkers. In a subset of subjects the full time course will be analysed to generate kinetics, based on the reactogenicity observed. All selection processes will involve the External Advisory Board of the BIOVACSAFE project who are international experts in the field of Systems Biology and vaccine safety. Where certain post immunization events are recorded (e.g. fever, injection site reaction) a full time course analysis across the event will be analysed within those subjects, acting as their own controls. Biomarkers that are significant according to previously published criteria for gene expression (e.g. Pulendran et al., 2010) will be selected for advancing into the animal models WP2, and into later stage clinical confirmatory trials.

Subsequently, we will prepare for at least two **Confirmatory Phase IV clinical trials.** We will recruit approximately 300 subjects to each trial, to power analysis sets to confirm biomarkers identified in the Training studies, using only one or two vaccines selected on the basis of the results obtained from the Training studies according to the analytic methodologies available in the statistical and systems biological expertise of partners in the project.

If successful, these biomarkers could be used in early stage clinical trials to optimize selection of vaccine candidates with a profile that will be unlikely to generate worrisome safety signals once they are in generalized use.

7.2 Summary of Study Design

The first three "training" studies of which this is one will have the **same schedule of assessments, study objectives and endpoints**. Each study will use a different licensed vaccine that is prototypical representative of a class of vaccine used in a particular target population.

In this study:

- "Engerix B" represents an **adjuvanted** **subunit vaccine** given to a population of healthy adults that have **no immunity** to that disease, in the context of **priming** and **booster** doses.

The study is single centre, partially blind (subject and laboratory), placebo controlled and randomised.

Subjects will receive three doses of the vaccine according to the SmPC. We will measure the clinical events (recorded adverse events), physiological responses (heart rate, blood pressure (BP), temperature, injection site), metabolic responses (serum concentration of metabolites and metabolic gene expression in whole blood), innate immune responses (cytokine levels and whole blood gene expression) after doses #1 and #3; and adaptive immune responses (serum antibody and antigen-specific cellular responses) at various time points after immunisation.

Subjects will be admitted to the clinical centre one day before doses #1 and #3 to allow stabilisation of their physiology, and to avoid background variations due to diet, exercise, alcohol intake, sleep patterns etc. This is especially important given the small sample size.

At each blood draw time point, full physiological parameters (including temperature, heart rate, BP and other physiological measurements) will be obtained and the injection site will be observed with any redness or swelling measured and recorded. Standardized diary cards will be used to collect solicited and unsolicited adverse event data at each blood draw time point. In-between outpatient visits 3, 4, 5 and 6, subjects will record their temperature if they experience any adverse events and measure, with a ruler, any skin reactions at the site of injection. Samples of blood (plasma, serum and PBMCs) will be collected for analysis and processing using protocols already in place. Subjects will also have blood obtained for standard pathology markers (haematology, chemistry) as well as acute phase proteins.

In this study we will seek to identify preliminary biomarkers associated with reactogenicity, or lack of reactogenicity for subsequent confirmation in larger clinical trials conducted as outpatients.

7.3 Profile of Study Drugs

ENGERIX B – Hepatitis B (rDNA) vaccine, adsorbed (HBV)

7.4 Potential Risks and Benefits

Please refer to the SmPC. The vaccine will be administered according to its UK SmPC, as a suspension for injection (20µg/1ml) of 1 mL (pre-filled syringe) administered intra-muscularly in the upper arm (deltoid region).

The SmPC details that Engerix B is indicated for active immunisation against hepatitis B virus infection in individuals who are seronegative to the Hepatitis B virus. It states that optimal protection is achieved if three doses (each of 1 ml) are administered at months 0, 1 and 6. In this study we will be using the vaccine as a prototypical representative of the class of adjuvanted vaccines administered to seronegative subjects, and we will intensively study the physiological responses to the first (priming) and third (booster) doses.

According to the SmPC, Engerix B is contra-indicated in individuals with known hypersensitivity to any component of the vaccine (excipients: sodium chloride, disodium phosphate dehydrate, sodium dihydrogen phosphate; Hepatitis B antigen produced in yeast cells) or in subjects who have exhibited hypersensitivity following previous Engerix B administration. Very common (≥ 10%) adverse reactions observed following immunisation with Engerix B, based on a total of 5329 doses, include: pain and redness at injection site, fatigue, and mood irritability.

7.5 Rationale For Study Design

7.5.1 Rationale for doses and schedule

The study will use the dose and immunisation schedule of Engerix indicated in the SmPC as the purpose of this study is to characterise clinical events, physiological and metabolic responses, and innate and adaptive immune responses following **priming** and **boosting** immunisation with products used in accordance with the UK SmPC. The placebo will be the same volume of physiological saline administered by the same route.

Subjects will be intensively studied as inpatients after immunisations #1 and #3 only, and will be given immunisation #2 during an outpatient visit. The selection of doses #1 and #3 allows us to intensively study physiological responses to the first priming dose in immunologically naive subjects, and delaying the second intensive evaluation to the booster dose #3 at 6 months allows any physiological changes from the previous immunisations to subside. Subjects will still receive the second dose at month 1 to ensure we mimic usual clinical practice, and so that subjects have the best chance to achieve protective immunity from participation. The placebo will be the same volume of physiological saline administered by the same route.

7.5.2 Rationale for Study Endpoints

The characterization of early immune response events following immunization (days 0-3, 7-28 for each dose) by gene expression and multiplex cytokine/cellular response analysis (systems biology approach) have been used to successfully identify biomarkers of inflammation and immune response/efficacy. This has been done in a

predictive and reproducible way by immunizing relatively small numbers of healthy subjects, for example with live viral Yellow Fever vaccine (Querec et al. 2009) or adjuvanted subunit malaria vaccines (Vahey et al., 2010). This opens-up the novel possibility to conduct intensive and highly focused, but relatively small clinical trials early-on in the development of novel vaccine technologies to identify biomarkers that may be predictive of safety signals that may only become apparent in subsequent larger scale clinical testing or during post marketing surveillance.

At this stage it is not possible to define which genes will be analysed, and which metabolites, cytokine and acute phase proteins will be measured in serum as these analysis sets are continuously evolving as technology and knowledge advances.

Blood will be taken for DNA to genotype subjects. However at this stage we will not conduct genotyping. Should specific biomarkers or clinical events be recorded, we may analyse the DNA for genetic markers to identify new variants associated with responses to vaccines, for validation in future clinical trials with larger numbers. All samples will be link-anonymised and no genetic data will be made available to subjects or their medical attendants. It is unlikely that the small sample size will identify rare variants, but as vaccine reactogenicity is not infrequent (up to 30% may experience some side effects) we may identify common traits. Also our biobank will provide a highly characterised set of samples to undertake future research should larger studies identify genetic traits associated with reactogenicity.

7.5.1 Blinding

The aim of these first studies will be to act as training studies to generate exploratory data sets. The studies will be partially-blinded in that the subject and laboratory will be blind as to treatment allocation. Specific laboratories will perform pre-defined assays on pre-defined clinical samples. Clinical study staff will be able to allocate samples from each treatment group to the correct laboratory for analysis as each laboratory may analyse only a subset of samples. Placebo samples will always be included and the laboratory will be blinded as to allocation of subjects to vaccine or placebo group. Once a laboratory has completed their assays and their database has been locked the results will be unblinded and used in the integrated systems biology analysis to design the subsequent Confirmatory Trials which will be double-blind.

8 STUDY OBJECTIVES AND ENDPOINTS

8.1 Exploratory Study Objectives

The purpose of this protocol is to generate an **exploratory training set** of data that will be analysed by integrated systems biology approach, for validation in subsequent clinical trials or in animal models. The dataset will broadly characterise:

1. Physiological responses at various time points after immunisation by measuring:
    a. Local and systemic vaccine-related clinical events.
    b. Physiological assessments: heart rate, temperature, blood pressure.
    c. Haematology (blood counts and ESR), Biochemistry (liver, renal and bone panels) parameters.

2. Metabolic, innate and adaptive immune responses including:
    a. Innate immune activation detected by global gene expression in whole blood
    b. Metabolism detected by metabolite concentrations in serum
    c. Adaptive immunity determined by serum anti-hepatitis B IgG titre
    d. Innate and adaptive immune activation detected by gene pathway activation in whole blood
    e. Metabolism detected by metabolic gene expression and pathway activation in whole blood
    f. Immune activation detected by:
        i. Concentration of selected inflammatory soluble mediators in serum including:
            1. chemokines and cytokines
            2. acute phase proteins
        ii. PBMC cytokine secretion, proliferation or surface markers in response to *in vitro* antigen stimulation
3. Genotype of subjects
4. Correlations in changes in innate immune activation and metabolism with adverse events, haematology and biochemistry panels, genotype and physiological assessments

We will biobank all samples for the duration of the BIOVACSAFE programme so that we can selectively analyse different samples and different time points depending on the results generated, principally from the gene expression analysis of whole blood.

8.2 **Study Endpoints** (study variables measured)

The exploratory data set will include numerous study variables which will be analysed by integrated systems biology approach. Not all samples and not all time points will be initially analysed: an iterative approach is followed whereby putative biomarkers that are identified (principally in the gene expression data) will be further studied by measuring correlated variables such as immune responses, clinical events or serum / cellular responses. Samples will be biobanked during the project duration to allow further analyses that may be initiated by observations from the linked clinical studies. Once the study objectives have been achieved samples will either be destroyed, entered into a registered biobank, or ethical approval sought for subsequent use.

The study variables will include:

1. Frequency of local and systemic vaccine-related clinical events at all time points from vaccination up to last study visit.
2. Change from pre-immunisation baseline values in heart rate, temperature, blood pressure at all time points from time of immunisation up to last study visit.

3. Change from pre-immunisation baseline values in haematology (blood counts and ESR), biochemistry (liver, renal and bone panels) parameters at selected time points from time of immunisation up to last study visit.
4. Change from pre-immunisation baseline values in global gene expression measured on whole blood samples at selected time points from time of immunisation up to last study visit
5. Change from pre-immunisation baseline values in metabolite concentrations in serum samples at selected time points from time of immunisation up to last study visit
6. Change from pre-immunisation baseline values in serum anti-hepatitis B IgG titre in serum samples at selected time points from time of immunisation up to last study visit
7. Change from pre-immunisation baseline values in metabolic gene expression and pathway activation measured on whole blood samples at selected time points from time of immunisation up to last study visit
8. Change from pre-immunisation baseline values in concentration of selected cytokines and acute phase proteins in serum samples at selected time points from time of immunisation up to last study visit
9. Change from pre-immunisation baseline values in PBMC cytokine secretion, proliferation or surface markers in response to in vitro antigen stimulation at selected time points from time of immunisation up to last study visit
10. Fold increase in serum hepatitis B IgG titre at selected time points after immunisation compared with pre-immunisation baseline.
11. Genotype of subject

9 SUBJECT SELECTION AND WITHDRAWAL CRITERIA

9.1 Population base

Healthy male and female volunteers aged between 18 and 45 years.
There will be 24 subjects randomised in the study:
1) Vaccine Group n = 20
2) Placebo Group, n = 4.

9.2 Inclusion criteria

1. Healthy male or female subjects aged 18-45 years inclusive.
2. The subject is, in the opinion of the investigator, healthy on the basis of a physical examination, medical history, vital signs, and the results of routine laboratory tests with no active disease process that could interfere with the study endpoints.
3. Body Mass Index ≥18.5 and <29.5
4. The subject is able to read and understand the Informed Consent Form (ICF), and understand study procedures.
5. The subject has signed the ICF.
6. The subject has not previously received a vaccine for Hepatitis B or contracted hepatitis B infection.

7. The subject is seronegative to Hepatitis B as confirmed at screening by assessments of sAb, sAg, and cAb.
8. Available for follow-up for the duration of the study.
9. Agree to abstain from donating blood during and for three months after the end of their participation in the study, or longer if necessary.
10. If heterosexually active female of child bearing potential, willing to use an effective method of contraception with partner (any hormonal contraception e.g. oral contraceptive pill, progesterone only pill; intrauterine device; injectable or implanted contraceptive; condoms incorporating spermicide if using these; physiological or anatomical sterility) from 30 days prior to the first vaccination until 3 months after the last, and willing to undergo urine pregnancy tests prior to vaccination and blood pregnancy test at screening and final follow up.
11. The subject has venous access sufficient to allow blood sampling as per the protocol.

9.3 Exclusion criteria

1. Significant dietary restrictions (e.g. vegan, lactose intolerant, but vegetarian acceptable) or life-threatening food allergies (e.g. anaphylaxis-related nut allergies).
2. Pregnant or lactating at any point during the study from screening to final follow up.
3. Known hypersensitivity to any component of the vaccine (excipients: sodium chloride, disodium phosphate dehydrate, sodium dihydrogen phosphate; Hepatitis B antigen produced in yeast cells) or subjects who have exhibited hypersensitivity following previous Engerix B administration; or known hypersensitivity to any other hepatitis B vaccine.
4. Presence of primary or acquired immunodeficiency states with a total lymphocyte count less than 1,200 per mm^3 or presenting other evidence of lack of cellular immune competence e.g. leukaemias, lymphomas, blood dyscrasias, or patients receiving immunosuppressive therapy (including regular use of oral, inhaled, topical or parenteral corticosteroids).
5. Use of any immune suppressing or immunomodulating drugs within 6 months of Visit 1 (screening).
6. Regular use of non-steroidal anti-inflammatory drugs (by any route of administration including topical) within 6 months of Visit 1 (screening) considered by the study physician as likely to interfere with immune responses.
7. Receipt of a vaccine within 30 days before visits 2 or 7, or at any time between visits 2 and 6, or between visit 7 and the final visit. Requirement to receive another vaccine within 30 days before visits 2 or 7, or at any time between visits 2 and 6, or between visit 7 and the final visit.
8. Presence of an acute severe febrile illness at time of immunisation.

9. History of alcohol, narcotic, benzodiazepine, or other substance abuse or dependence within the 12 months preceding Visit 1.
10. Currently participating in another clinical study with an investigational or non-investigational drug or device, or has participated in a clinical trial within the 3 months preceding Visit 1.
11. Any condition that, in the investigator's opinion, compromises the subject's ability to meet protocol requirements or to complete the study.
12. Receipt of blood products or immunoglobin, or blood donation, within 3 months of screening.
13. Unable to read and speak English to a fluency level adequate for the full comprehension of procedures required in participation and consent.
14. An average weekly alcohol intake that exceeds 14 or 21 units per week for females and males, respectively (1 unit = 12 oz or 360ml of beer; 5oz or 150ml of wine; 1.5oz or 45ml of distilled spirits), or unwilling to stop alcohol consumption for each treatment period during the study.
15. Currently smokes in excess of 5 cigarettes/day or equivalent use of tobacco (within the last 6 months of screening), or subjects unwilling to refrain from smoking or are unable to abide by Surrey CRC restrictions.
16. Consumes excessive amounts, defined as greater than 4 servings (1 serving is approximately equivalent to 120mg caffeine) of coffee, tea, cola, or other caffeinated beverages/food per day.

No waivers from the Protocol will be allowed.

9.4 Discontinuation and Withdrawal of subjects from the study

Any subject may voluntarily discontinue participation in this study at any time.

The investigator may also, at his or her discretion, discontinue the subject from participating in this study at any time, and must do so if any of the following criteria are met:

- Positive pregnancy test at any visit.
- Concomitant use of any medication which may interfere with study outcome and study medication throughout the study.

The reason for termination will be recorded in the case report form (CRF). A subject may withdraw (or be withdrawn) from the study prematurely for the following reasons:

- Withdrawal of consent
- Adverse event (AE section must be completed)
- Protocol deviation
- Lost to follow-up

- Other (must be specified)

If a subject discontinues/withdraws prior to randomisation to study treatment (Visit 2) they will be considered as a screen failure. Screen failures must be replaced to ensure that 24 subjects are randomised with equal gender allocation.

A follow-up visit should be performed if premature discontinuation/withdrawal following randomisation at Visit 2 takes place.

All participants who withdraw or are withdrawn from the study before completion of visit 8 will be replaced.

10 CLINICAL STUDY PROTOCOL

There will be 11 visits during the study: nine outpatient visits and two residential visits of seven days/six nights. Visit 1 will be screening, visits 2 & 7 will be the inpatient sessions and visits 3, 4, 5, 6, 8, 9, 10 & 11 will be outpatient follow-up visits.

The study will commence after a favourable opinion has been obtained from the Ethics Committee. Written informed consent will be obtained after a subject is informed of the nature, significance, implications and risks of the study and prior to the commencement of any study specific procedures.

10.1 Study Procedures

Visit 1: Screening Visit (Day – 28 to -2)

Visit 1 will take place up to 28 days prior to the immunisation/residential session (Visit 2). Subjects will arrive at the Surrey CRC having been advised to abstain from alcohol for 48 hours, and the procedures listed in Table 1 will be performed.

Visit 2 & 7: Residential Visits with Immunisation (doses #1 and #3 days -1 to +5)

The subjects will be admitted on the morning of Day -1 and be required to remain in the centre until the evening of Day +5. Throughout this time the subjects will be monitored by clinical staff and Adverse Events will be recorded continually.

On admission and throughout the stay the procedures listed in Table 2 will be performed. The day of immunisation will be day 0 to calculate days / weeks before or after immunisation.

Subjects presenting with an illness, ongoing injury, or who are febrile at visit 2 will have visit 2 delayed until the clinical event has resolved completely as this would adversely affect the biomarker assays. For each group, if the subjects are still eligible to continue on the study then they will be randomised to one of two treatment groups:

A) Engerix B Group n = 20
B) Saline Placebo Group, n = 4 .

Immunisations will be staggered such that the last subject may be immunised up to 60 minutes after the first. All subsequent procedures will be performed relative to dosing at set time points. A window of ±15 minutes is acceptable at each time point. Procedures should be conducted in the following order: vital signs, blood samples and assessment of clinical events. Assessments made during the sleep period will require the subjects to be woken.

Visits 3, 4, 5 and 6 (cycle 1: priming); 8, 9, 10 and 11 (cycle 2: boosting): Follow Up Visits (dose #1 and #3 of vaccine days +7, +14, +21 & +28)

These outpatient follow-up visits will occur on specific days relative to immunisation.

A visit window of ±1 day is acceptable for each visit but should be avoided wherever possible and used only in a situation where a sample would otherwise be lost. Outside of this the visit procedures should still be undertaken and all samples collected, but will be recorded as a protocol deviation. The procedures listed in Table 1 will be performed.

10.2 Duration of Study Participation

Each subject would be participating in the study for a maximum of 7 months.

10.3 Study Restrictions

For the duration of the study subjects will maintain a normal lifestyle in terms of e.g. diet, sleep, activity and exercise. Whilst subjects are outpatients there will be minor restrictions but no monitoring of their typical lifestyle. When the subjects are residential in the clinical unit they will experience as normal a lifestyle as is possible but under regulated conditions.

Subjects will be required to comply with the following for the duration of the study:

10.3.1 Dietary Restrictions

Diet will be unrestricted but on Outpatient Visit days subjects will be requested to have their normal breakfast (noting the restrictions on caffeine). Whilst residential at Surrey CRC, all food and beverages will be provided and subjects will only be able to consume what has been supplied. Diet will be standardised for nutrient content each residential day.

10.3.2 Activity Restrictions

No strenuous exercise will be permitted whilst subjects are resident at Surrey CRC. For 48 hours prior to admission to visit 2 and 7 subjects will be asked to limit their exercise.

10.3.3 Alcohol Restrictions

Subjects will be advised to refrain from drinking alcohol for 48 hours prior to all visits and no alcohol is permitted during the Residential Visit. On any other days subjects are allowed to consume 3 (men) or 2 (women) units of alcohol per day [NOTE: 1 unit is equivalent to a half-pint (220mL) of beer or 1 (25mL) measure of spirits or 1 small glass (125mL) of wine].

10.3.4 Caffeine Restrictions

Subjects will be advised to not consume any beverages containing caffeine, including tea, coffee, chocolate, or cola type drinks for 8 hours prior to, or during, an Outpatient visit or the Residential visit. At all other times during the study, subjects will undertake not to consume more than 3 caffeine-containing beverages per day from screening until the final treatment visit. Only non-caffeinated beverages will be supplied during the Residential visit.

10.3.5 Smoking Restrictions

Surrey CRC operates a strict non-smoking policy, and subjects will not be permitted any nicotine containing products whilst attending/resident in the unit.

Subjects will be required to state when they consent to participate in the study that they agree to abide by these instructions.

10.4 Prohibited Medications and Non-Drug Therapies

Subjects must not receive another vaccine within 30 days of visit 2 or 7, or at any time between visits 2 and 6, or between visit 7 and the final visit. Subjects may receive necessary vaccines in the window between completing visit 6 and up to 30 days before visit 7.

The study vaccine must not be administered at any time during the study period following visit 1.

Subjects are permitted to use oral contraceptives, vitamins and minerals throughout the study period, and the occasional use of paracetamol and over the counter drugs, including those for hay fever or allergies.

Paracetamol or non-steroidal anti-inflammatory drugs (e.g. Brufen) will not be administered during the inpatient stay except for the treatment of an adverse event that is graded severe, as these may interfere with primary and secondary study endpoints of innate immune responses and study clinical assessments.

Regular use of non-steroidal anti-inflammatory drugs or any use of immunosuppressing or immunomodulating drugs is prohibited during the course of the study. If a subject takes any of the above medications they will be withdrawn from the study.

If subjects are prescribed any new medications (including creams, ointments, inhalers) or if they purchase any medications ‘over the counter’ (including herbal remedies), for example from the pharmacy, supermarket or health food shop, they must report the name of the drug(s)/herbal preparation(s), the dose and the dates, duration of treatment at their next visit and reason for use which will subsequently be recorded in the source document and CRF. Subjects will be questioned at each assessment visit about the use of concomitant medication in the period since the last assessment visit.

11 STUDY MEDICATION

11.1 Treatment Plan

GROUP A: The study medication will be ENGERIX B – adjuvanted Hepatitis B virus. The study medication will be supplied as a 1 mL suspension for injection (20μg/1mL) pre-filled syringe.

GROUP B: The placebo control will be 1 mL physiological saline administered with the same gauge needle as the vaccine.

11.2 Preparation, Administration and Dosage of Study Medication

11.2.1 Preparation Instructions

Engerix B is supplied in a pre-filled syringe ready for use.

Placebo control: 1 mL physiological saline should be drawn into a 1 mL syringe.

11.2.2 Route of administration

The vaccination or placebo will be administered intra-muscularly into the deltoid muscle, according to the UK SmPC.

11.2.3 Dose and Dosing Schedule

All subjects will receive three immunisations according to the UK SmPC. Immunisations #1 and #3 will be administered intra-muscularly in accordance with the schedule in Table 2 and described in section 10.1; immunisation #2 will be administered on Day 28 at no fixed time.

11.3 Packaging and Labelling

As the study medication has a marketing authorisation in the UK, is being used within the terms of that marketing authorisation and will not be repackaged for use in the study, reduced labelling will be used. The following particulars will be added to the original container, but will not obscure the original labelling:
i) University of Surrey
ii) Study reference code, investigator and study participant number.

11.4 Storage and Accountability of Drug Supplies

The vaccine should be stored at +2°C to +8°C (in a refrigerator).

Simplified accountability records will be kept, to capture the batch number of the product dispensed on a study-specific prescription form, filed in a study folder to permit retrospective verification if this was necessary.

11.5 Return of Clinical Supplies

Any unused vaccines will remain in their original packaging. After study medication accountability has completed they will be disposed of by discard into the standard clinical waste system.

11.6 Responsibilities

- Named trained NMC study nurses will be responsible for ensuring that the vaccine is securely maintained.
- Named trained NMC study nurses will be responsible for ensuring that an accurate record of vaccine issued and returned is maintained.
- Vaccine quality issues will be reported to the manufacturer by the PI.

11.7 Compliance

Vaccines will be administered at the clinical site by trained NMC study nurses.

11.8 Treatment Assignment Procedures

At screening (visit 1), all subjects screened will be allocated the lowest available screening number. The screening numbers available will be 8001 – 8999.

At visit 2, once continuing eligibility has been determined, the subject will be randomised into a study group and allocated the lowest available subject number. The subject numbers available will be 201 – 299. To allow for replacement subjects a parallel replacement randomisation list with numbers 1201 to 1299 will be generated. Corresponding numbers will be randomised to the same group e.g. 202 and 1202 would be in the same group, and replacement subjects will be allocated the corresponding number to the subject they are replacing e.g. 202 would be replaced by 1202.

11.8.1 Randomisation

Randomisation codes will be provided in sealed envelopes. A program to generate a randomisation list linking subject numbers to treatment group will be produced by an independent statistician using a computer-generated algorithm. An independent member of staff will then use this program to produce the randomisation list. All eligible subjects that pass screening will be randomised as one cohort into one of the treatment groups. Consideration of equal numbers of males and females will be made in the randomisation and wherever possible attempts will be made to have equal numbers of men and women in each vaccine and placebo group.

11.8.2 Blinding/Unblinding

The aim of these first studies will be to act as training studies to generate exploratory data sets. The studies will be partially-blinded in that the subject and laboratory will be blind as to treatment allocation. Specific laboratories will perform pre-defined assays on pre-defined clinical samples. Clinical study staff will be able to allocate samples from each treatment group to the correct laboratory for analysis as each laboratory may analyse only a subset of samples. Placebo samples will always be included and the laboratory will be blinded as to allocation of subjects to vaccine or placebo group. Once a laboratory has completed their assays and their database has been locked the results will be unblinded and used in the integrated systems biology analysis to design the subsequent Confirmatory Trials which will be double-blind. It is crucial to the project that the unblinded data is made available as soon as possible

so that the parameters to be measured in the confirmatory trials can be selected in a timely manner.

12 STUDY DATA PARAMETERS

12.1 Laboratory Assessments

Blood samples will be collected at the time points specified in Tables 1 and 2. Standard laboratory tests, including chemistry, haematology, and urinalysis panels, will be performed. Pregnancy tests will be performed for female subjects at screening, prior to vaccine/placebo administration and at the end of the study.

From Visit 2 onwards, blood samples for transcriptomics, metabolomics, proteomics and serum immunology will be collected at time points specified in the study flow charts.

'Serum Save'

Several of the parameters being investigated will be analysed in serum. A 'serum save' strategy will be used to ensure that enough serum is collected at each time point for each of the required parameters to be measured. The 'serum save' strategy applies to the following sample types: acute phase proteins, metabolomics, chemokines & cytokines, serum immunology samples, and 'biobank' samples. In practice, when a blood sample is collected the extracted serum will be separated into serum aliquots. These will be stored and made available for analysis within the 5-year Biovacsafe project time period.

Biobank

At every time point a serum sample is required, enough blood will be collected to extract the required volume of serum for the parameter(s) being measured plus 1ml of additional serum, which will be entered into a biobank. The biobank will be used to analyse other parameters which become of interest during the study and which would contribute to the aims of the Biovacsafe project. Every effort will be made to collect only the minimum volume of blood from participants. However, on occasion it is possible that slightly more serum will be extracted from the blood samples than is required. Should this occur, the remaining serum will also be added to the biobank.

Genotyping

One blood sample for DNA genotyping will be collected for each subject prior to vaccine/placebo administration, for the purposes described in section 7.5. The blood sample will be anonymised so that it will not be possible to link the results of the genotyping back to the subject that provided the sample, other than through the investigator site.

12.1.1 Study Blood Volume

The total volume taken during the study is detailed in Table 3, below. Female subjects will have an additional 4 mL of blood taken for two blood pregnancy tests taken at Visits 1 and 11. The total volume taken will be approximately 1390.5 mL for males and 1394.5 mL for females.

Table 3. Volumes of blood collected during the study for a male subject

Parameter	Visit 1 Screening Blood volume (ml)	Visits 2 & 3 or 7 & 8 Blood volume (ml)	Visits 4 – 6 or 9 - 11 Blood volume (ml)	Visits 2 – 6 or 7 – 11 Total volume (ml)	Visits 1 – 11 Total volume (ml)
Safety blood samples[a]	12.5	81	25.5	106.5	225.5
RNA		67.5	7.5	75	150
Genotype		9		9[a]	9
PBMC		120	72	192	384
Serum (immunology, acute phase proteins, metabolomics, chemokines, cytokines, biobank)		236	75	311	622
TOTAL	**12.5**	**513.5**	**180**	**693.5**	**1390.5**

[a]Only on visit 2

12.1.2 Urinalysis

Urinalysis will be performed by dipstick to evaluate protein, glucose, pH, white and red blood cells. Microscopy may be performed if the dipstick is clinically significant.

12.2 Vital signs

Vital signs include: diastolic and systolic blood pressure measurement following five minutes in a supine position, heart rate and oral temperature. They will be obtained at the screening visit, during the treatment period and at the follow-up visit.

12.3 Measurement of temperature & injection site reactions

Subjects will be provided with a thermometer and a ruler to measure respectively the temperature in the event of adverse events and the diameter of local adverse events of induration or erythema at injection site. Such measurements should be taken following discharge from Surrey CRC at visit 2 and between follow up visits 3, 4, 5 and 6. The measurements will be recorded in the subject diary cards.

12.4 Methods and Timing for Assessing and Recording Study Data Parameters

Laboratory safety tests (biochemistry, haematology, urinalysis) and vital sign measurements will be performed at scheduled time points throughout the study. These procedures may also be performed at unscheduled time points if considered clinically necessary by the investigator. The detailed outline of the study procedures can be found in Tables 1 and 2.

13 SAFETY MONITORING

All subjects will receive a study information card that indicates their participation in the study and details contact information for the study site.

13.1 Specification of Safety Parameters

13.1.1 Laboratory and Other Safety Assessment Abnormalities Reported as AEs and SAEs

Any abnormal laboratory test results (haematology, clinical chemistry or urinalysis) or other safety assessments (e.g. vital signs measurements), including those that worsen from baseline, and are felt to be clinically significant in the medical and scientific judgement of the investigator, are to be recorded as AEs or SAEs.

However, any clinically significant safety assessments that are associated with the underlying disease, unless judged by the investigator to be more severe than expected for the subject’s condition, are **not** to be reported as AEs or SAEs.

13.1.2 Pregnancy

In accordance with MHRA regulations, the Marketing Authorisation Holder should record all instances of 'drug exposure during pregnancy' in their pharmacovigilance program and follow up on all reports relating to pregnancies at the expected due date. These reports do not need to be expedited if there is no adverse drug reaction.

Any pregnancy that occurs during study participation must be reported as an individual case safety report to the marketing authorisation holder. The PI will confirm the withdrawal of the subject from the study.

13.1.3 Definition of an AE

- Any untoward medical occurrence in a patient or clinical study subject, to whom a medicinal product has been administered and which does not necessarily have a causal relationship with this treatment.

  Note: An AE can therefore be any unfavourable and unintended sign (including an abnormal laboratory finding), symptom, or disease (new or exacerbated) temporally associated with the use of a study medication whether or not related to the study medication. For marketed medicinal products, this also includes failure to produce expected benefits (i.e. lack of efficacy), abuse or misuse.

  Events meeting the definition of an AE include:

  • Exacerbation of a chronic or intermittent pre-existing condition including either an increase in frequency and/or intensity of the condition

  • New conditions detected or diagnosed after study medication administration even though it may have been present prior to the start of the study

  • Signs, symptoms, or the clinical sequelae of a suspected interaction

  • Signs, symptoms, or the clinical sequelae of a suspected overdose of either study medication or a concomitant medication (overdose *per se* will not be reported as an AE/SAE).

  Events that **do not** meet the definition of an AE include:

  • Medical or surgical procedure (e.g. endoscopy, appendectomy); the condition that leads to the procedure is an AE.

  • Situations where an untoward medical occurrence did not occur (social and/or convenience admission to a hospital).

  • Anticipated day-to-day fluctuations of pre-existing disease(s) or condition(s) present or detected at the start of the study that do not worsen.

  • The disease/disorder being studied or expected progression, signs, or symptoms of the disease/disorder being studied, unless more severe than expected for the subject's condition.

13.1.4 *Definition of a SAE*

Definition of Serious Adverse Events will follow UK National Research Ethics guidance "SAFETY REPORTING (Research other than CTIMPs)" in which in other research other than CTIMPs, a serious adverse event (SAE) is defined as an untoward occurrence that:

(a) results in death;
(b) is life-threatening;
(c) requires hospitalisation or prolongation of existing hospitalisation;
(d) results in persistent or significant disability or incapacity;
(e) consists of a congenital anomaly or birth defect; or
(f) is otherwise considered medically significant by the investigator.

13.2 Evaluating and Recording Adverse Events

The investigator or designee is responsible for detecting, documenting and reporting events that meet the definition of an AE or SAE. These must be recorded in the source adverse event form and/or CRF.

For all adverse events, the following must be assessed and recorded on the adverse events page of the CRF:

a) Description of AE
b) Start date and time
c) End date and time
d) Severity i.e. mild, moderate, severe (see below)
e) Relationship to study medication(s) – completed by study physician
f) Action taken with study medication
g) Action/ Treatment required, e.g. paracetamol
h) Outcome
i) Seriousness (see section on Serious Adverse Events)

SAEs and AEs will be collected from the time of the first immunisation is given until the last visit. However any SAEs assessed as related to study participation (e.g. study medication, protocol-mandated procedures, invasive tests, or change in existing therapy) will be recorded from the time a subject consents to participate in the study up to and including the last visit.

13.3 Intensity of Event

The intensity of an adverse event is defined as follows:

Mild Transient symptoms, requiring no treatment, no interference with subject's daily activities, easily tolerated.

Moderate Marked symptoms, moderate interference with the subject's daily activities, usually ameliorated by simple therapeutic measures.

Severe Considerable interference with the subject's daily activities, requires intensive therapeutic intervention, incapacitating.

The term **severe** is a measure of **intensity**: thus a severe AE is not necessarily **serious.** For example, nausea of several hours duration may be rated as severe, but may not be clinically serious.

13.4 Relationship to study product

The relationship of each adverse event to the study medication must be recorded by a medically qualified member of staff as one of the following scale:

Definitely Not related The AE is judged to be clearly and incontrovertibly due only to extraneous causes (for example, disease, environment) definitely not associated with the test drug being given and does not meet the criteria for any other drug relationship listed.

Probably Not related In general, this category is applicable to an AE which meets the following criteria (it certainly must meet the first two criteria):

1. It does not follow a reasonable temporal sequence from the drug administration.
2. It may readily have been produced by the subject's clinical state, environmental or toxic factors, or other modes of therapy administered to the subject.
3. It does not follow a known pattern of response to the suspected drug.
4. It does not reappear or worsen when the drug is re-administered.

Possibly Related This category applies to those AEs in which the connection with the test drug administration appears unlikely but cannot be ruled out with certainty. An AE may be considered as possibly drug related if, or when:

1. It follows a reasonable temporal sequence from administration of the drug.
2. It may have been produced by the subject's clinical state, environmental or toxic factors or other modes of therapy administered to the subject.
3. It follows a known pattern of response to the suspected drug.

Probably Related This category applies to those AEs which are considered, with a high degree of certainty, to be related to the test drug. An AE may be considered as probably drug related if:

1. It follows a reasonable temporal sequence from administration of the drug.
2. It cannot be reasonably explained by the known characteristics of the subject's clinical state, environmental or toxic factors, or other modes.

3. It disappears or decreases on cessation or reduction in dose (there are important exceptions when an AE does not disappear upon discontinuation of the drug, yet drug-relatedness clearly exists).

4. It follows a known pattern of response to the suspected drug.

5. It reappears upon re-challenge.

Definitely Related This category applies to those AEs which are considered to be definitely related to the test drug. An AE may be considered as Definitely related if:

1. There is evidence of exposure to the test drug.

2. It follows a reasonable temporal sequence from administration of the drug.

3. It cannot be reasonably explained by the known characteristics of the subject's clinical state, environmental or toxic factors, or other modes.

4. The AE is more likely explained by the test drug than by any other cause.

5. Dechallenge is positive.

6. Re-challenge (if feasible) is positive.

7. The AE shows a pattern consistent with previous knowledge of the test drug or test drug class.

13.5 Expectedness of SAEs

For this study, an adverse reaction is 'unexpected' if its nature and severity are not consistent with the information about the study medication in question, set out in the SmPC. A medically qualified member of staff must assign expectedness.

13.6 Reporting of Serious Adverse Events

Reporting of Serious Adverse Events will follow UK National Research Ethics guidance "SAFETY REPORTING (Research other than CTIMPs)" in which an SAE occurring to a research participant should be reported to the main REC where in the opinion of the Chief Investigator the event was:

- Related – that is, it resulted from administration of any of the research procedures, and
- Unexpected – that is, the type of event is unexpected as defined in section 13.5.

Timelines will be as follows (taken from NRES "SAFETY REPORTING (Research other than CTIMPs)"):

What	Who	When	How	To Whom
**SAE**	Chief Investigator (CI) or sponsor.	Within 15 days of the CI becoming aware of the event.	SAE report form for non-CTIMPs, available from NRES website.	Main REC for the study.
**Urgent safety measures**	Chief Investigator or sponsor. *Or (ii) exceptionally by local Principal Investigator (PI).*	(i) Immediately.<br><br>(ii) Within 3 days.	(i) By telephone.<br><br>(ii) Notice in writing setting out the reasons for the urgent safety measures and the plan for further action.	Main REC for the study. REC Co-ordinator will acknowledge within 30 days. *If notified by PI, relevant local REC should also be informed.*

In addition, the MHRA Yellow Card scheme, which allows suspected side effects to licensed medicines and vaccines to be reported online will be used according to the scheme guidance.

13.7 Progress reporting

Progress reporting will follow UK National Research Ethics guidance "PROGRESS REPORTING (Research other than CTIMPs)":

Type	Who	When	How	To Whom
Progress reports	To be submitted by sponsor, sponsor's legal representative or Chief Investigator (CI). Must always be signed by CI.	Annually (starting 12 months after the date of the favourable opinion) *Main REC may exceptionally request more frequent reports.*	Annual progress report form (non-CTIMPs), available from NRES website.	Main REC for the study.
Declaration of the conclusion or early termination of the research	Sponsor or CI.	Within 90 days (conclusion). Within 15 days (early termination). *The end of the study should be defined in the protocol.*	End of study declaration form, available from the NRES website.	Main REC for the study.

Summary of final report	Sponsor or CI.	Within one year of the conclusion of the research.	No standard format. The summary should include information on whether the study achieved its objectives, the main findings and arrangements for publication or dissemination including feedback to participants.	Main REC for the study.

13.8 Treatment and Follow-up of Adverse Events

All adverse events must be documented and followed up until the event is either resolved or adequately explained, even after the subject has completed his/her study treatment.

In the case of any SAE, the subject must be followed up until clinical recovery is complete and laboratory results have returned to normal or until progression has been stabilised. This may mean that follow-up will continue after the subject has completed the clinical study and that additional investigations may be requested by the sponsor.

SAEs that are spontaneously reported by a subject to the investigator after study completion and considered by the investigator to be caused by the study medication with a reasonable possibility should be handled in the same manner as for SAEs reported during the study.

In the event of unexplained clinically abnormal laboratory test values, the tests should be repeated immediately and followed up until the results have returned to within the range of normal and/or an adequate explanation of the abnormality is given.

If a clear explanation is established, it should be recorded on the CRF.

13.9 Overdose of Study Medication

If the subject has taken, accidentally or intentionally, any study medication administered as part of the protocol, and exceeded the dose prescribed by the protocol, the investigator or the reporting physician is to decide whether a dose is to be considered an overdose, but the Sponsor has the authority to upgrade the determination, if deemed appropriate. Before upgrading a report, it is necessary for the Sponsor to communicate with the investigator and reach a consensus.

13.9.1 Management of Overdose

The UK SmPC states that cases of accidental administration of more than the recommended dose of Engerix B have been reported. Amongst these cases, the adverse events reported were similar to those reported with normal vaccine

administration. No specific management of Engerix B overdose is required, other than those related to any specific adverse event that may occur.

14 STATISTICAL CONSIDERATIONS AND ANALYTICAL PLAN

14.1 Responsibility for Analysis

The analysis of the data obtained from this study will be the responsibility of the Surrey CRC, the systems biology groups of University of Surrey, Chalmers University and MPIIB Berlin, assisted by other partners of the BIOVACSAFE consortium. Additional integrated systems biology analysis may be undertaken by specialist laboratories and collaborators.

14.2 Justification of Sample Size

This study will utilize an integrated systems biology approach to assess acute and longitudinal responses of all measured variables to immunization to identify exploratory putative biomarkers. These may then be further characterised and confirmed in subsequent trials and animal models. As such, a variety of –omic technologies will be used to identify novel biomarkers in an iterative fashion where the biobank of samples is repeatedly accessed; as the identity of these markers and the magnitude of response are unclear, it is not possible to use existing data to perform power calculations and determine an appropriate sample size. In addition the samples and data from all the clinical training studies (involving 5 different vaccines) will be combined into the integrated analysis, altogether with the data from the two larger trials that will follow (around 600 subjects) and the various animal models.

A previous study utilized a systems biology approach to detect early gene ‘signatures’ to predict immune responses in individuals immunized with the yellow fever vaccine YF-17D (Querec et al., 2009). With a sample size of 15 it was possible to detect distinct signatures that predicted the neutralizing antibody response with up to 100% accuracy. For this study a sample size of n = 20 has been selected for each exploratory training study as this should allow for significant changes in –omic profiles to be detected. The placebo groups from all the different studies will be pooled to produce a comparable group of n = 20.

14.3 Definition of Study Completion

Study completion is defined as the date the last subject completes the final visit in the study.

14.4 Definition of Criteria for Termination of the Study

Study termination is defined as a permanent discontinuation of the study due to unanticipated concerns of safety to the study subjects arising from AEs recorded during the study that are definitely related to the protocol, and are not present in the SmPC; or availability of other new data (pharmacokinetic, pharmacodynamic, efficacy, biologic etc) arising from clinical or preclinical studies with this study drug. A study may be paused during review of newly available preclinical/clinical safety, pharmacokinetic, pharmacodynamic, efficacy, or biologic data, or other issues of

interest or potential concern prior to a final decision for continuation or termination of the study.

14.5 Analysis of Clinical Events and Exploratory Endpoints after immunisation

All subjects who receive a dose of study medication will be included.

All AEs will be coded using MedDRA prior to database lock. Adverse events will be analysed, irrespective of their causal relationship, by treatment group. The terms mentioned in the tabulations will be the WHO system organ class and preferred terms.

For Serious Adverse Events meeting the definition of section 13.6, tabulations will provide by treatment group, the number of subjects exposed, the number of subjects with at least one Serious Adverse Event, the number of subjects with at least one Serious Adverse Event by organ system and preferred term. Separate tables will be provided, if relevant, for SAEs leading to withdrawal from study.

Listing of AEs will be carried out by the Surrey CRC Statistics Team. All AEs will be listed by treatment and subject and will give the following details: description of event, the code, date of onset, duration, severity, relationship to treatment, whether serious, outcome and corrective treatment if any.

No formal statistical analysis of clinical events or any of the endpoints will be performed other than as part of the integrated systems biology analyses

15 STUDY DOCUMENTATION ADMINISTRATION

15.1 Source documents and CRF/ eCRF

In case of direct entry, the CRF will be considered as source documentation for the following items:

Body weight & height, BMI, vital signs, blood sampling times, dates and times of visits and assessments.

All other evaluations that are reported in the CRF must be supported by appropriately signed identified source documentation related but not limited to:

Subject identification, last participation in the study, medical history, laboratory assessments and AEs.

CRFs are provided for each subject, and all data related to the study will be recorded in these CRFs. The CRFs are to be completed at the time of the subject’s visit so that they always reflect the latest observations on the subjects.

The investigator must verify that all data entries in the CRFs are accurate and correct by signing the relevant pages. If certain information is not available, not applicable, not done or unknown, the clinical staff or investigator will enter the relevant abbreviation, i.e. NA to confirm that the data field has not been overlooked.

This also applies to subjects who fail to complete the study. If a subject withdraws from the study, the reason must be noted on the CRF. If a subject is withdrawn from the study because of a treatment-limiting adverse event, thorough efforts should be made clearly to document the outcome.

All forms should be typed or filled out using a black ball-point pen, and must be legible. All entries, corrections and alterations are to be made by the responsible investigator or her/his designee. With the exception of obvious mistakes, the corrections need to be commented. Corrections should be made in such a way that the original entry is not obscured. The corrected data should be entered, dated, and initialled by the investigator or his designee.

15.2 Monitoring

Monitoring will be in accordance with applicable regulations and standards of GCP.
When reviewing data collection procedures, discussion will include identification, agreement and documentation of data items for which the CRF will serve as the source document.
The monitor will review the CRFs, evaluate them for accuracy (including source data verification) and completeness, and return all forms with missing information and/or errors to the clinical staff or investigator for correction.
The monitor will supervise the study to ensure that the:

- Data are authentic, accurate, and complete
- Safety and rights of subjects are being protected
- Study is conducted in accordance with the currently approved protocol and any other study agreements, GCP and all applicable regulatory requirements

15.3 Access to Source Data Documents

The investigator will permit study–related monitoring, audits, IRB/IEC review, and regulatory inspections by providing direct access to source data/documents.

15.4 Data Handling and Record Retention

The investigator must maintain adequate records to enable the conduct of the study to be fully documented. The investigator should arrange for retention of the essential documents in the investigator's Study Master File for at least five years after the final study report has been signed. No study-related documents will be destroyed until receipt of written permission from the Sponsor.

Any difficulty in storing original documents should be discussed with the monitor prior to initiation of the study.

15.5 Subject Confidentiality and Data Protection

The investigator must ensure that subject's anonymity will be maintained. On CRFs or other documents, subjects should **not** be identified by their names, but by a screening number. The investigator should keep a separate enrolment log showing screening number, names and addresses. Documents such as subjects' separate written consent forms should be maintained by the investigator in strict confidence.

16 QUALITY CONTROL AND QUALITY ASSURANCE

To ensure compliance with GCP and all applicable regulatory requirements, the sponsor may conduct a quality assurance audit of the site records, and the regulatory agencies may conduct a regulatory inspection at any time during or after completion of the study. In the event of an audit or inspection, the investigator (and institution) must agree to grant the auditor(s) and inspector(s) direct access to all relevant documents and to allocate their time and the time of their staff to discuss any findings/relevant issues.

A study monitor appointed by the Sponsor is responsible for visiting the institution at regular intervals throughout the study to verify adherence to the protocol; completeness, accuracy, and consistency of the data; and adherence to ICH GCP and local regulations on the conduct of clinical research. The monitor is responsible for inspecting the CRFs and ensuring completeness of the study essential documents. The monitor should have access to subject medical records and other study-related records needed to verify the entries on the CRFs. The monitor will communicate deviations from the protocol, SOPs, GCP and applicable regulations to the investigator and will ensure that appropriate action designed to prevent recurrence of the detected deviations is taken and documented. The investigator agrees to co-.operate with the monitor to ensure that any problems detected in the course of these monitoring visits are addressed and documented.

17 CLINICAL STUDY PROTOCOL DEVIATIONS AND AMENDMENTS

Any ‘substantial’ protocol amendment(s) (meaning that it could have a significant impact on the safety or physical or mental integrity of the subjects, the scientific value of the study, the conduct or management of the study, the quality or the safety of the vaccine used in the study) must be submitted to the Independent Ethics Committee (IEC) prior to its implementation.

Amendments to exploratory endpoints, objectives, parameters and variables will not be considered substantial.

For non-substantial changes that do not affect safety or study validity e.g. an administrative change, IEC is not required to be notified. Non-substantial amendments will be reported to the IEC at the time of a subsequent substantial amendment.

In the case of changes consisting of urgent safety measures to protect the study subjects, the sponsor should inform the IEC as soon as possible after these measures have been implemented.

18 CONDITIONS FOR TERMINATING THE STUDY

Study completion is defined as the date the last subject completes the final visit in the study. Study termination is defined in section 14.4.

If, in the opinion of the investigator, the clinical observations or pharmacokinetic profiles in the study suggest that it may be unwise to continue, the investigator may terminate part of, or the entire study, after consultation with the sponsor, or the sponsor may terminate part of, or the entire study, for safety or administrative reasons. A written statement fully documenting the reasons for such termination will be provided to the IEC.

19 <u>ETHICAL AND REGULATORY REQUIREMENTS</u>

The study will be conducted in compliance with the protocol, principles of GCP, Data Protection Act and other regulatory requirements, as appropriate, and will abide by the principles of the 2008 revision of the Declaration of Helsinki.

19.1 Informed Consent

It is the responsibility of the investigator to obtain written informed consent from each subject participating in this study, after adequate explanation of the aims, methods, anticipated benefits, and potential hazards of the study. This includes obtaining the appropriate signatures and dates on the informed consent document prior to the performance of any study specific procedures. Subjects will be given written information outlining the study details given approval by the IEC. Any changes to the approved version of the information sheet/consent form must be approved by the IEC prior to its implementation, unless it is for urgent safety measures. A copy of the signed consent form will need to be given to the subject.

The investigator, or person under his responsibility, must also explain that the subject is completely free to refuse to enter the study or to withdraw from it at any time. The CRF for this study contains a section for documenting informed consent, and the investigator, or person under his responsibility, must complete it appropriately.

19.2 Independent Ethics Committee (IEC)

This protocol and any accompanying material provided to the subjects (such as the information sheet or description of the study used to obtain informed consent) will be submitted by the investigator, or person under his responsibility, to the appropriate IEC. Approval from the committee must be obtained in writing before starting the study and the approval letter must reference which documents were reviewed and approved.

Any required changes will be forwarded to the IEC for their approval. Written approval of the revised documents should also be obtained from the IEC. Depending upon the exact changes, written approval of the revised documents may not be required prior to the commencement of the screening process.

The IEC must provide a copy of their membership list, and a list of names of those members present at the meeting when the study was reviewed. The IEC must also have provided a copy of their constitution, and a signed statement indicating that it complies with GCP, that will be kept on file at Surrey CRC.

19.3 Annual/Final Reports

The sponsor will notify the IEC within 90 days of the end of the study. If the study is terminated prematurely, this reporting timeframe will be reduced to 15 days from the termination of the study.

The study report will be provided to the IEC within one year of completion of the study, as defined in Section 18 of this protocol.

The Sponsor will be responsible for submitting annual reports to the IEC.

20 FINANCE AND INSURANCE

Prior to starting the study, the Sponsor will secure funding for the conduct of this study. Each of these agreements will include the financial information agreed upon by the parties.

Reimbursement, indemnity and insurance are also addressed in the Project and Grant Agreements for the Innovative Medicines Initiative project BIOVACSAFE proposal number 115308.

21 PUBLICATIONS

Before recruitment the study will be registered with clinicaltrials.gov (http://clinicaltrials.gov/) website.

All publications and communications arising from this study will comply with the Project and Grant Agreements for the Innovative Medicines Initiative project BIOVACSAFE proposal number 115308.

22 REFERENCES

Pulendran, B, Li, S and Nakaya,, HI (2010). Systems vaccinology. *Immunity,* 33, 516 – 29.

Querec, TD, Akondy, RS, Lee, EK, Cao, W, Nakaya, HI, Teuwen, D, Pirani, A, Gernert, K, Deng, J, Marzolf, B, Kennedy, K, Wu, H, Bennouna, S, Oluoch, H, Miller, J, Vencio, RZ, Mulligan, M, Aderem A, Ahmed, R, Pulendran, B (2009). Systems biology approach predicts immunogenicity of the yellow fever vaccine in humans. *Nat Immunol*, 10, 116 – 25.

Vahey, MT, Wang, Z, Kester, KE, Cummings, J, Heppner, DG Jr, Nau, ME, Ofori-Anyinam, O, Cohen, J, Coche, T, Ballou, WR, Ockenhouse, CF (2010). Expression of genes associated with immunoproteasome processing of major histocompatibility complex peptides is indicative of protection with adjuvanted RTS, S malaria vaccine. *J Infect Dis,* 15, 580 – 9.
